# Baculovirus Insecticides in Latin America: Historical Overview, Current Status and Future Perspectives

**DOI:** 10.3390/v7052230

**Published:** 2015-04-30

**Authors:** Santiago Haase, Alicia Sciocco-Cap, Víctor Romanowski

**Affiliations:** 1 Instituto de Biotecnología y Biología Molecular (IBBM), Departamento de Ciencias Biológicas, Facultad de Ciencias Exactas, Universidad Nacional de La Plata, CONICET, La Plata 1900, Argentina; E-Mail: shaase@biol.unlp.edu.ar; 2 Instituto de Microbiología y Zoología Agrícola (IMYZA), Instituto Nacional de Tecnología Agropecuaria (INTA), Castelar 1712, Argentina; E-Mail: sciocco.alicia@inta.gob.ar

**Keywords:** viral biopesticides, baculovirus, Latin America, crop protection

## Abstract

Baculoviruses are known to regulate many insect populations in nature. Their host-specificity is very high, usually restricted to a single or a few closely related insect species. They are amongst the safest pesticides, with no or negligible effects on non-target organisms, including beneficial insects, vertebrates and plants. Baculovirus-based pesticides are compatible with integrated pest management strategies and the expansion of their application will significantly reduce the risks associated with the use of synthetic chemical insecticides. Several successful baculovirus-based pest control programs have taken place in Latin American countries. Sustainable agriculture (a trend promoted by state authorities in most Latin American countries) will benefit from the wider use of registered viral pesticides and new viral products that are in the process of registration and others in the applied research pipeline. The success of baculovirus-based control programs depends upon collaborative efforts among government and research institutions, growers associations, and private companies, which realize the importance of using strategies that protect human health and the environment at large. Initiatives to develop new regulations that promote the use of this type of ecological alternatives tailored to different local conditions and farming systems are underway.

## 1. Introduction

### 1.1. Biological Control as an Essential Component of Integrated Pest Management Strategy

In the past few decades, appreciation of the negative impacts of insecticide usage on the environment and health led to efforts directed towards a reduction in chemical control of pests and weeds. Many countries have become stricter in the regulation of pesticide manufacture, registration, and use. These policies have resulted in higher costs, and shortage of these tools in some cases. On many occasions, the behavior of the pests themselves demanded a change in control strategies as resistance to insecticides has become a frequent phenomenon [1,2,3].

These practices require a combined strategy known as integrated pest management (IPM), aiming at a significant reduction or elimination of chemical pesticides. A major contribution to this type of strategies is the use of biological control methods, including natural enemies and pathogens specific for the insect pests.

In principle, biological control can be long-term due to persistence of the pathogens in the environment [4]. The natural enemies and entomopathogens applied intentionally may establish themselves in the pest population and contribute to long-term crop protection. Pathogens including fungi, nematodes, bacteria, and viruses can effectively control pests when applied artificially as insecticides [5,6].

### 1.2. Baculoviruses: Molecular Biology, Ecology and Application as Biopesticides

Among the insect viruses found in nature, those belonging to the baculovirus family (*Baculoviridae*) were considered for the development of most commercial viral biopesticides [7,8,9,10].

Members of this family are regarded as safe for vertebrates and, to date, no cases of pathogenicity of a baculovirus to a vertebrate have been reported [11,12,13,14,15,16]. Moreover, their host-specificity is usually very narrow and often limited to single insect species.

Baculoviruses are insect-specific, enveloped viruses with circular, supercoiled double-stranded DNA genomes in the range of *ca.* 80–180 kbp [17]. More than 600 baculoviruses have been isolated from Lepidoptera (butterflies and moths), Hymenoptera (sawflies), and Diptera (mosquitoes) [18]. The name “baculovirus” is derived from the rod-shaped, nucleocapsids (Latin “*baculum*”: stick) which are 230–385 nm in length and 40–60 nm in diameter [17]. The virions are enveloped and two phenotypes have been recognized: occlusion derived virus (ODV) and budded virus (BV). These two types of virions contain the same genome but differ in the morphogenesis and composition of their envelopes and their functions in the virus life cycle. Their stabilities in the environment, as well as their infectivities to the target insect, are extremely different.

The ODV are enclosed in a paracrystalline protein (polyhedrin or granulin) matrix forming an occlusion body (OB). This structure is quite resistant to diverse environmental conditions and therefore facilitates persistence and horizontal transmission of the disease in nature. ODV consist of one or more nucleocapsids enclosed in a single lipoprotein membrane envelope. Different baculoviruses are characterized by OBs containing either a single virion with a single nucleocapsid, multiple virions with a single nucleocapsid each or multiple virions containing bundles of several nucleocapsids. The surface of the OBs is covered by an envelope or calyx composed of protein with a large proportion of carbohydrate [17].

The morphology of OB was used to define two major groups of *Baculoviridae*: Nucleopolyhedrovirus (NPVs) and Granulovirus (GVs). OBs of NPVs, also known as polyhedral inclusion bodies (PIBs) or simply polyhedra, are about 0.6–2 μm in size, large enough to be seen under a light microscope [19], and their major occlusion protein is called polyhedrin. OBs of GV, known as capsules or granules, are oval with diameters in the range of 0.2–0.4 μm, and the major protein is granulin.

More recently, the sequencing of many complete baculoviral genomes and a more detailed phylogenetic analysis of viruses and their natural hosts was used to define four genera: *Alphabaculovirus* (lepidopteran NPV), *Betabaculovirus* (lepidopteran GV), *Gammabaculovirus* (hymenopteran NPV), and *Deltabaculovirus* (dipteran NPV) [18,20,21].

**Figure 1:**
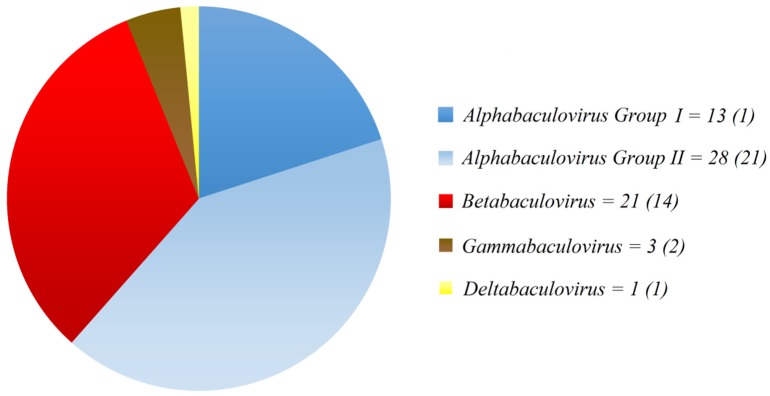
*Baculoviridae*. The numbers of putative species in each of the four genera are based on the species accepted by the International Committee on Taxonomy of Viruses, ICTV (numbers in parentheses) plus other baculoviruses that are not recongnized as species yet, but the information published to date suggest their inclusion in the near future as separate species according to the species demarcation criteria adopted by ICTV [18,20,21]. Redundant genomes were excluded and the more recently sequenced *Erinnyis ello* granulovirus [22], *Agrotis segetum* NPV-B [23], *Spodoptera frugiperda* GV [24], and *Pseudoplusia includens* SNPV [25], among others, were added. As an alternative to complete genome information, species can be defined following the demarcation criteria set forth in [20]. The graph shows the *Alphabaculovirus* genus divided in groups I and II, based on the active fusogenic protein present in the BV.

The proportion of species described for each genus can be appreciated in Figure 1. By and large, *Alphabaculovirus* is the taxon with many more species [18] than the other three genera and the type species for *Alphabaculovirus* is AcMNPV (*Autographa californica multiple nucleopolyhedrovirus*).

**Figure 2:**
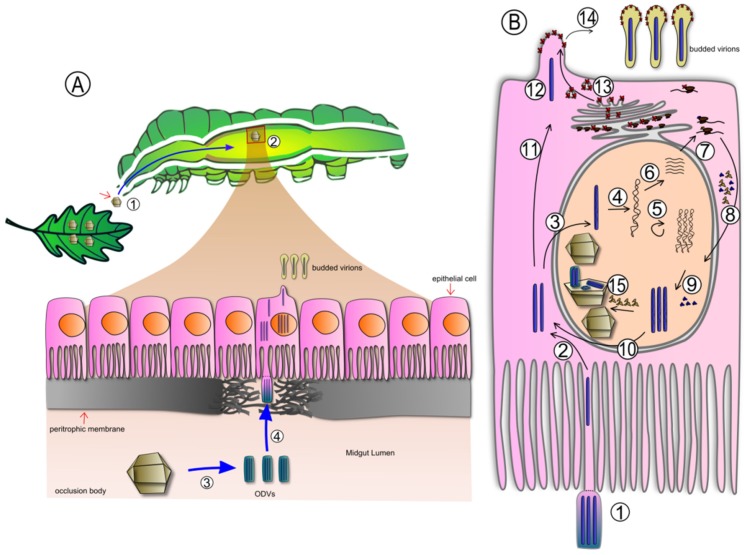
Baculovirus infectious cycle. (**A**) Cross sectional schematic of an insect larva. A baculovirus occlusion body (OB) ingested with contaminated food starts a new infectious cycle (1). When OBs pass through the foregut and reach the alkaline midgut the proteinaceous matrix is dissolved (2), releasing ODV (3). The peritrophic membrane is degraded by virus and host encoded enzymes present in the OB (4), allowing the ODV to enter the cell; (**B**) Representation of the virus replication cycle. ODV enters the cell by fusion with epithelial cell microvilli (1), releasing nucleocapsids (NC) into the cytoplasm (2). NC may enter the nucleus (3), disassemble and release the genome (4). Then early genes are transcribed (6) and translated (7). Some of the proteins translocate into the nucleus (8), take part in genome transcription/replication, NC and virion assembly (9). In the first stages of viral infection, NC is transported to the cytoplasm (10), approaches the basolateral cell membrane (CM) (11) and emerges as budded virus (BV) (12) in the spots where the viral envelope fusion protein (EFP) (14) accumulates using the secretory pathway (13). In the very late stages of infection, NC are enveloped in the nucleus and occluded in the polyhedral shaped protein matrix (OB) (15) (adapted from [27], copyright 2013, The Authors).

The natural cycle of infection of insect larvae by AcMNPV is schematized in Figure 2. Caterpillars ingest polyhedral that contaminate their food. The alkaline environment of the midgut triggers the dissolution of polyhedra (OB) and the release of ODV into the midgut lumen [26].

Once released, ODV face the barrier of the peritrophic membrane (PM), a lattice of chitin, mucopolysaccharides and proteins that separates food from midgut tissue [28]. The PM lattice has pore sizes ranging from 21 to 36 nm in diameter [29], so that small particles, such as degradative enzymes, can pass freely through the lattice as part of the digestive process, but the passage of larger particles, such as pathogens, is restricted. ODV must damage the PM to gain access to the midgut epithelium. In order to do this, some baculoviruses encode a class of metalloproteases called enhancins, which cleave mucin-like proteins bridging chitin strands in the PM lattice [30]. It has ben reported that enhancins are co-occluded with ODVs in the OB matrix [31] or are present on ODV surfaces [32], and catalyze the disruption of the PM after the OB is dissolved. Not all the baculoviruses encode enhancins, and there are other viral and host-encoded factors that degrade the PM [33]. In these cases, the addition of enhancing proteins from other baculoviruses may contribute to a faster PM degradation and reduced significantly the time of action of the virus [34]. The ODV enter the midgut cell after fusion with epithelial cell membrane. The virions are uncoated and the nucleocapsids (NC) enter the nucleus, where viral genes are expressed in a controlled manner. The first type of progeny that emerges from the primary infection consists of BV, which spreads the infection to other tissues. In the very late stages the production of infectious BV is reduced, and the newly assembled NC acquire an envelope in the nucleus and are finally occluded by polyhedrin, which makes up more than 95% of the OB. Up to 10^10^ polyhedra are produced per larva, frequently accounting for more than 30% of the dry weight of a caterpillar [35]. However, in some baculovirus-host systems infection cannot spread beyond the midgut epithelium. In [36], Passarelli thoroughly reviews the barriers that baculovirus have to overcome in order to establish efficient systemic infections.

Fibrillar structures composed mostly of the very late protein P10 accumulate in the cell in association with microtubule and are involved in the proper assembly of polyhedral envelope (PE) [17]. These structures have been implicated in the disintegration of the host cells [37]. In the final stages of infection, viral-encoded enzymes, chitinase, and cathepsin, are essential for the breakdown of the host cuticle and the final liquefaction of the larvae [38]. Polyhedra released from the dead larvae remain in the environment and can be horizontally transmitted to other caterpillars when they ingest OBs present on leaves [39]. Vertical transmission via contamination of eggs may also play a role in spreading the virus [40].

The first well-documented introduction of a baculovirus to the environment which resulted in effective suppression of a pest occurred in the 1930s, when, along with a parasitoid imported from Europe to the USA and Canada to control spruce sawfly *Diprion hercyniae*, a NPV specific for this insect was introduced accidentally [41,42]. Since then, no control measures have been required against *Diprion hercyniae*. Moreover, the NPV now occurs in populations of *Neodiprion sertifer* and *Diprion hercyniae* in North America. The introduction of an exogenous baculovirus is unusual. More commonly, two alternative strategies of pest management are used: infested areas are sprayed with highly concentrated baculovirus insecticide formulations to control the pest as quickly as possible, or sprayed with lower concentrations of baculovirus, leading to the establishment of the virus for several insect generations [43]. Most of the examples refer to baculoviruses isolated from the local insect host and are one of the causes for the fluctuations in population dynamics in particular areas.

## 2. Examples of Baculovirus Control Programs in Latin America

The most significant cases of insect pest control programs based on baculovirus in Latin America are described in the following sections. Special consideration should be given to the context of each case, since the feasibility and success of a program relies on the commitment of governments, extension agencies, research groups, farmers and general public information. Although the use of baculoviruses for the protection of agricultural annual crops, fruit orchards and forests has not been as extensive as it was expected, there are a number of successful examples that are summarized in the following sections and that will hopefully be expanded as studies on new baculovirus-host systems progress. We shall concentrate on case studies in Latin America.

**Figure 3:**
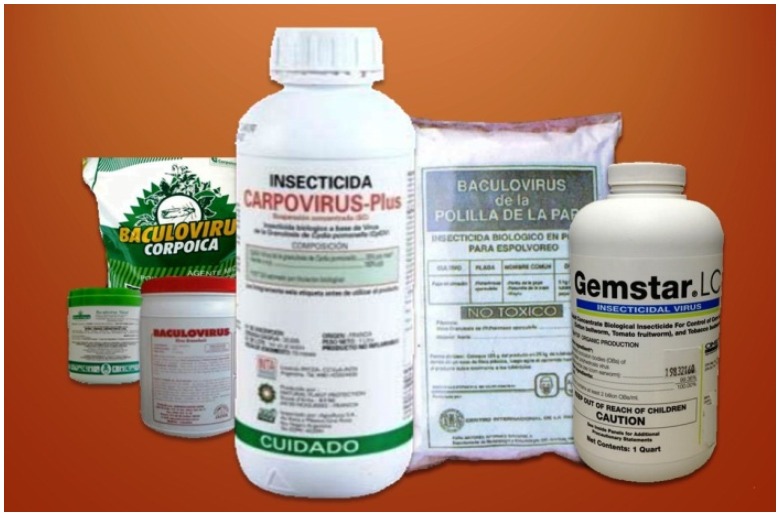
Some baculoviral pesticides commercialized in Latin America.

A summary of baculovirus-based products commercialized and mostly produced in Latin America can be found in Table 1, and Figure 3 is a sample of these commercial products.

**Table 1:** Examples of baculovirus-based products commercialized in Latin America.

Virus	Host	Crops	Product	Country	Producer company
*Anticarsia gemmatalis* MNPV	*Anticarsia gemmatalis*	Soybean	Baculo-soja ^1^, Baculovirus Nitral ^2^, Coopervirus SC ^3^, protégé ^4^, Multigen ^5^	Brazil	Nova Era Biotecnología Agrícola ^1^, Nitral Urbana ^2^, COODETEC ^3^, Milenia ^4^, EMBRAPA ^5^
*Autographa californica* MNPV + *Spodoptera albula* NPV	*Autographa califórnica Trichoplusia ni Pseudoplusia includens Heliothis virescens Spodoptera exigua Estigmene acrea Plutella xylostella*	Alfalfa, vegetable crops	VPN-ULTRA	Guatemala	Agricola El Sol
*Spodoptera sunia* NPV	*Spodoptera* spp.	Vegetables	VPN 82	Guatemala	Agricola El Sol
*Cydia pomonella* GV	*Cydia pomonella*, *C. pomonella*, *Grapholita molesta*	Apple, pear, walnut Apple, peach	Carpovirus Plus ^6^ Madex ^7^ Carpovirusine ^6^ Madex Twin ^7^	Argentina ^6^ Argentina ^7^ Chile ^6^ Uruguay ^7^	NPP-Arysta Life Science ^6^ Andermatt Biocontrol ^7^
*Erinnyis ello* GV	*Erinnyis ello*	Cassava ^8^ Rubber trees ^9^	Baculovirus erinnyis ^8,9,10^	Brazil ^8^ Colombia ^9^ Colombia ^10^	Empresa de Pesquisa Agropecuária e Extensão Rural de Santa Catarina S.A. ^8^ BioCaribe SA ^9^ CORPOICA ^10^
*Helicoverpa zea* SNPV	*Heliothis and Helicoverpa* spp.	Maize, tomato, cotton and tobacco	Gemstar ^11^ HzNPV CCAB ^12^	Mexico ^11^ Brazil^12^	Certis USA ^11^ AgBiTech Australia ^12^
*Helicoverpa armigera* NPV	*Heliothis and Helicoverpa* spp.	Tomato, sweet pepper, maize, soybean, tobacco, vegetable crops	Diplomata ^13^ Helicovex ^14^	Brazil ^13, 14^	Koppert ^13^ Andermatt Biocontrol ^14^
*Phthorimaea operculella* GV	*Phthorimaea operculella Tecia solanivora*	Potato	Baculovirus Corpoica ^15^ PTM baculovirus ^16, 17^	Colombia ^15^ Peru ^16^ Costa Rica ^17^	CORPOICA ^15^ SENASA Peru ^16^ INTA Costa Rica ^17^
*Phthorimaea operculella* GV + *Bacillus thuringiensis*	*Phtorimaea operculella Tecia solanivora Symmetrischema tangolias*	Potato	Matapol Plus ^18^ Bacu-Turin ^19^	Bolivia ^18^ Ecuador ^19^	PROINPA Foundation ^18^ INIAP, Ecuador ^19^
*Spodoptera exigua* NPV	*Spodoptera exigua*	Tomato, chili, eggplant	SPOD-X LC	Mexico	Certis USA—SUMMIT AGRO Mexico
*Spodoptera frugiperda* MNPV	*Spodoptera frugiperda*	Maize, sorghum	-	Brazil	EMBRAPA (in development)

*Note:* the superscripts ^(1-19)^ are included for disambiguation in order to associate the biopesticide (product), the producer company and the country or countries where the particular biopesticides are applied. In particular, two products identified with the same designation, *e.i.* PTM baculovirus ^16, 17^ are produced by different organizations in two different countries Peru^16^ and Costa Rica ^17^.

### 2.1. AgMNPV for the Control of the Velvetbean Caterpillar in Soybean Crops

The velvetbean caterpillar, *Anticarsia gemmatalis* (Lepidoptera: Noctuidae), is one of the major foliage feeding pests of legume crops in South America, affecting mainly soybean fields. In Brazil, the severe impact on soybean crops was reduced in the past with organochlorine and organophosphorus chemical insecticides. The control of *A. gemmatalis* required two insecticide applications during one season, which raised the concern for the impact on both environment and human health. This led to the development of an IPM program [44] based on the periodic monitoring of the pest and the application of minimal dosages of nonpersistent chemical pesticides only when insect pest exceeded thresholds based on assessment of defoliation levels, pest populations, and presence of pathogens, mainly the fungus *Nomuraea rileyi* (Farlow) Samson.

In the 1970s, a nucleopolyhedrovirus of *A. gemmatalis* (AgMNPV) was isolated in different regions of Brazil [45,46]. Initial field experiments with the AgMNPV revealed its potential as a biopesticide in soybean IPM programs. In 1980/1981 and 1981/1982, a pilot use of AgMNPV was conducted under the coordination of Dr. Flavio Moscardi (EMBRAPA) on 21 farms in the southern states of Brazil. These trials, conducted with virus produced by collecting dead larvae in the fields, were accompanied by the training of extension workers in the application of AgMNPV. These extension workers played a critical role, providing a direct connection with the farmers, monitoring the fields and collecting data that allowed evaluating the performance of the biopesticide [47].

The subsequent success of the AgMNPV program (reviewed in [48,49]) was due in large part to the collaborative work of EMBRAPA researchers and extension workers to convince farmers of the benefits of the use of AgMNPV in the biological control of the pest. This was achieved through the organization of outreach activities focused on showing and discussing the results obtained. In addition, farmers that participated in the pilot phase were helpful to convince other farmers to try the AgMNPV-based insecticides.

Results of the pilot phase were satisfactory, and EMBRAPA and official and private extension services decided to implement a program for the use of AgMNPV in the 1982/83 season. For this phase, AgMNPV was produced in *A. gemmatalis* larvae reared on artificial diet. From this point, dead larvae from AgMNPV treated fields were collected to provide inoculum to spray other areas during the same season or to store inoculum for the next season. The model of AgMNPV field production was established as the most convenient method to obtain large amounts of OBs at a low cost.

In 1986 a wettable powder formulation based on AgMNPV was developed [47,50] and at the end of the 1980s Embrapa started to negotiate contracts with private companies interested in producing and commercializing the biopesticide. The commercialization of the AgMNPV by five private companies expanded its use to about one million hectares in 1990–1991 and two million hectares by 2002–2003 [51,52].

Despite the efforts deployed by Embrapa and other research institutes to develop and improve mass-production of AgMNPV under controlled laboratory conditions, the private companies that tried to implement this methodology lost economic competitiveness against those that used the more primitive field production methodology [47]. This was due to the high cost of labor, disposable rearing containers and components of the artificial diet. Field production demanded a large amount of manpower, since the dead larvae were harvested manually, and had the disadvantage that the quality and quantity of infected larvae was dependent upon the natural prevalence of the host insect that could vary from year to year. Harvesting of infected insects in the field involved from 200 to 300 larval pickers per day, and resulted in the collection of hundreds of kilograms of dead larvae. In the most productive AgMNPV season (2002/2003), about 45 tons of AgMNPV-killed caterpillars were collected, representing more than 2.0 million hectares-equivalents of biopesticide [53].

More problems appeared when manual larvae harvesting was replaced by an automated collection procedure in which the plants were shaken over drop cloths. This method led to a poor quality product because the material included other insects, debris, and *A. gemmatalis* that were not in the final stages of the virus infection cycle. The final product was of lower infectivity and caused problems during application.

The limitations of AgMNPV field production stimulated studies aiming at improving the laboratory production of the biopesticide. The artificial diet components for *A. gemmatalis* rearing were evaluated and their cost was reduced about seven-fold and the AgMNPV laboratory production rates on the revised diet were comparable to those of AgMNPV from larvae collected in the field [54]. The private company Codetec adopted a laboratory production method, with a potential to treat 1.8–2.0 million hectares per year. However, this company discontinued this initiative due to the reduced demand for AgMNPV for the reasons that are discussed in the following paragraph.

The reduced demand of AgMNPV resulted from the advent of no-till agricultural systems in Brazil, which caused soybean growers to adopt the common practice of applying herbicides before sowing. Unfortunately, many farmers acquired technological packages sold by companies that included the combined application of chemical insecticides and herbicides. This practice led to the decline of natural enemies, [49,55]. In this context, other insects that usually caused little damage in soybean crops became important pests [48,56]. The use of AgMNPV in Brazil was reduced substantially, and presently this virus is applied to about 200,000 hectares per year in recent last seasons [57].

AgMNPV was also used in soybean fields in Paraguay since the early 1990s with good results [58]. The biopesticide is still used in Paraguay in about 100,000 hectares per year. Field trials assays were also carried out in Argentina and Colombia, although in these countries pest control programmes based on AgMNPV have not been still established.

More recently researchers at Mexico’s National Institute for Agriculture, Forestry and Livestock Research (INIFAP) began studying the use of the virus for control of the velvetbeen caterpillar in soybean in the north of the country. As a result, AgMNPV is now used regularly over an area of 15,000 ha of soybean, with major reductions in the use of chemical insecticides, yielding the additional benefit of maintaining higher population densities of natural enemies and lower incidence of secondary pests [59]. Large quantities of the biopesticide are produced using an in-field-production system.

### 2.2. CpGV for the Control of the Codling Moth in Apple and Pear Orchards

*Cydia pomonella* granulovirus (CpGV) was originally isolated from larvae collected in Chihuahua, Mexico [60,61]. Since its description and development as a bioinsecticide, it has provided an effective alternative for the control of the codling moth in integrated and organic pome fruit and walnut production in several countries [62,63]. In Argentina, the first field trials were conducted during the early 1980s, in pear and apple orchards in the province of Mendoza (western Argentina). Since 1987, the work was continued by the Institute of Agricultural Microbiology and Zoology (IMYZA, INTA), resulting in the development of CpGV-INTA-503 as an active ingredient and its registration for experimental use. Since 2000, subsequent development was conducted through material and technology transfer agreements signed by INTA and Natural Plant Protection (France)—Arysta Life Science for the registration of the commercial product Carpovirus, and AgroRoca S.A. (Argentina) for its commercialization. Since 2002, a new formulation (Carpovirus Plus^®^) has been available in the market and successfully used for the management of codling moth in the main production areas [64]. In 2007, other CpGV based product, Madex^®^ (Andermatt Biocontrol—Switzerland), was registered by Agricheck SRL.

**Figure 4:**
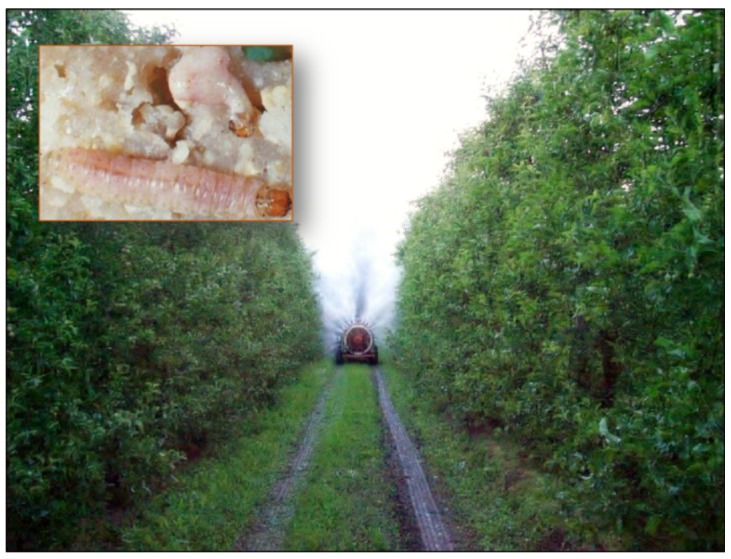
Application of Carpovirus Plus^®^ in apple orchards in Río Negro, Argentina. Inset: *C. pomonella* larvae infected by CpGV (Image kindly provided by Graciela. Quintana; Instituto de Microbiología y Zoología Agrícola, Instituto Nacional de Tecnología Agropecuaria (IMYZA.INTA), Castelar, Argentina).

In Alto Valle del Río Negro, the largest region of pear and apple production in Argentina, highly satisfactory results were achieved when the virus was applied as the only control method or in combination with conventional chemicals at doses of 10^13^ OBs/hectare at intervals of 8–10 days between treatments (Figure 4).

In organic production, with a 14 day interval between treatments, no detectable levels of damage were apparent at time of harvest, when the biopesticide was combined with a pheromone-based mating disruption technique. Annually, thousands of tons of fresh fruit treated with the virus enter the demanding international market [65,66]. The growing use of CpGV in walnut orchards impacted significantly on small farms in the valleys of Catamarca and La Rioja (Northwestern Argentina), where this safe and effective product doubled or even tripled the amount of fruit produced. The virus applications were performed every 10–12 days, with doses of 10^13^ OBs/hectare alone or in combination with conventional chemical insecticides, such as azinphos-methyl, lambda-cyhalothrin and cypermethrin [67,68].

In 2005, after more than twenty years of use, evidence emerged on the existence of European *C*. *pomonella* populations resistant to commercial CpGV pesticides [69,70,71,72,73]. European CpGV products were based on a Mexican isolate (CpGV-M) first described in [60]. This finding stimulated the search and selection of new CpGV isolates [74,75,76,77]. In Argentina, after more than 10 years of sustained use of the virus, resistant CpGV-M populations have not been detected. However, in order to prevent or delay the expression of resistance in local pest populations, studies were initiated to evaluate the effectiveness of new isolates and the use of strategies which include the use of CpGV in combination with insecticides with minimal environmental impact (*i.e.*, methoxyfenozide, rynaxypyr). In parallel, research institutes are working in coordination with SENASA-Argentina (National Health and Food Quality Service) in the framework of the National Program for the Suppression of Codling Moth [66]. In addition, new isolates and formulations are being assayed to provide control of the oriental fruit moth, *Grapholita molesta* in peach [78].

In Chile, Carpovirusine^®^ was registered for commercial use by Arysta Life Science and more recently, Madex Twin was approved in Uruguay for the control of two pests, the codling moth and the oriental fruit moth. Finally, experimental field trials were also conducted recently in Mexico using 0.77–3.3 × 10^12^ OBs per hectare. Significant protection levels of of fruit were achieved indicating that low CpGV doses are effective for controlling the codling moth, as long as they are applied on the day of emergence of the larvae [79].

### 2.3. Phthorimaea Operculella GV for the Control of the Potato Tuber Moth Complex

The potato (*Solanum tuberosum* L., Solanaceae) is the third most important food crop in the world after rice and wheat in terms of human consumption. The global production of potato exceeds 300 million metric tons annually [80]. Although the potato originated in South America, this region has the lowest level of production (less than 16 million tons). However, for most small farmers in the Andes this crop remains a traditional food stuff, and is cultivated with other species of *Solanum* unknown to the rest of the world. There are over 4000 edible potato varieties, mostly found in the Andean region. In countries, such as Argentina, Brazil, Colombia, and Mexico, the commercial scale production of *Solanum tuberosum* is increasing.

Among the main insect pests, species belonging to the potato tuber moth (PTM) complex (Lepidoptera: Gelechiidae) cause severe damage worldwide [81,82]. Their larvae produce losses by mining the tubers in the field and during storage [83]. Originally an insect of the Andean region, the PTM, *Phthorimaea operculella* (Zeller) (Lepidoptera: Gelechiidae) has become an invasive potato pest globally [84]. Another tuber moth present in Peru, Bolivia and Colombia is *Symmetrischema tangolias*, and more recently, *Tecia solanivora* (Povolny) has invaded several countries in Central America.

A granulovirus (PhopGV) has been isolated from *P. operculella* in many countries in the world, and examined for their potential to control the pest [85,86,87,88,89,90,91].

In Peru, a PhopGV isolate has been developed as a microbial pesticide through an initiative of the International Potato Center (CIP). Virus infected larvae were ground and mixed with talc at a ratio of 20 larvae per kg talc and used as a suspension in 1 L of water. In addition, a dry product has been applied at a dose of 5 kg per ton of stored potatoes, providing high levels of control (*ca.* 95% mortality) [83,92]. The program was then established in Bolivia, Ecuador and Colombia [47]. In Bolivia, it was carried out by PROINPA (Fundación de Promoción e Investigación de Productos Andinos), and the bioinsecticide (Matapol^®^) was produced in a pilot plant with a capacity of 6 tons/year. The product was effective in the control of *P. operculella* but not against *S. tangolias*, and consequently, a new formulation containing PhopGV and *Bacillus thuringiensis* was developed [93] and is available in the market with the commercial name Matapol Plus^®^ in this country and as Bacu-Turicin in Ecuador [94].

It is well established that different PhopGV isolates vary in their activity against different populations of *P. operculella* and alternate hosts [95,96,97,98]. PhopGV is able to infect other species of Gelechiidae, such as *Tuta* (*Scrobipalpuloides*) *absoluta* (Meyrick) and *T. solanivora* [99,100,101,102,103], and with the aim to find isolates also effective against these species, there is a renewed interest in characterization and evaluation of new isolates in several countries, in order to develop new biopesticidal products. The Colombian Corporation for Agricultural Research (CORPOICA) conducted samplings of *T. solanivora* larvae in Colombia with the purpose of finding local virus isolates. As a result, five geographical granulovirus isolates from *T. solanivora* (named VG001, VG002, VG003, VG004, and VG005) were identified, and analysis by restriction endonuclease cleavage patterns revealed the presence of three different genotypic variants. Based on their DNA restriction patterns and biological activity, VG001 and VG005 isolates were selected for further analysis as potential biological control agents [104]. Mixtures of virus isolates showed a higher insecticidal activity compared to individual PhopGV isolates when applied to both *T. solanivora* and *T*. *absoluta*. This level of pathogenicity was maintained after numerous passages. The mixtures were about 3- to 25-fold (from 7.15 OBs/mm^2^ to 0.10 OBs/mm^2^) more pathogenic against *P. operculella* than the Peruvian isolate applied alone by surface contamination techniques. When tested on *T. solanivora*, they were between *ca.* two- and five-fold (from 12.29 OBs/mm^2^ to 1.25 OBs/mm^2^) more pathogenic than the isolate VG003 alone. A study of a biopesticide containing a mixture of various selected genotypes active against the target pests was conducted to develop a biopesticide effective against *P. operculella* and *T. solanivora* [105]. At present, a formulation of PhopGV, “Baculovirus CORPOICA” is the only baculovirus product registered in Colombia, and is recommended for the control of *T. solanivora* in stored potatoes.

Another PhopGV isolate collected from diseased *P. operculella* larvae collected in Costa Rica (PhopGV-CR1) was characterized. PhopGV-CR1 was highly pathogenic against its two indigenous hosts, although significant differences of up to four-fold were detected against *P. operculella* (LD_50_ = 17.9 OBs/mm^2^) and *Tecia solanivora* (Povolny) (Lepidoptera: Gelechiidae) (LD_50_ = 69.1 OBs/mm^2^). Serial passage of PhopGV-CR1 over four generations in *T. solanivora* resulted in an increase in its pathogenicity by about five-fold in three generations, suggesting a rapid adaptation to its alternate host [106]. The isolate was also evaluated under storage conditions, resulting in a decrease of damage of over 70% when compared with the untreated controls. In a prospective study, a French-Ecuadorean research team isolated some twenty different PhopGV from *P. operculella*, *T. solanivora*, and other gelechid species. More recently, in an effort to develop a viral biopesticide for the control of the Guatemala PTM *T. solanivora* they tested eight of these isolates and found a 14-fold difference in pathogenicity among them [107].

In Brazil, an indigenous PhopGV isolated from the PTM was characterized and evaluated against *P. operculella* and *T. absoluta* [108]. This isolate was formulated as a liquid suspension and evaluated alone, in mixtures with two commercial neem oil-based products (NeemAzal™ and DalNeem™, produced from the neem tree *Azadirachta indica*), and compared with a dry powder formulation of viral granules. High larval mortality (about 90%) was achieved when OBs and DalNeem™ (azadirachtin preparation) were applied together (10^4^ OBs/mL and 4 mg of azadirachtin/L). This combination resulted in ≥50% increase in efficacy compared with each of the components alone. A talc-based virus formulation resulted in 100% larval mortality at 5 × 10^8^ OBs/g, and provided a better control efficiency on PTM than an aqueous virus suspension. The PhopGV combined with DalNeem™ at low rates or formulated with talc powder appeared to represent a viable option for control of the PTM under storage conditions.

### 2.4. Spodoptera Frugiperda MNPV and GV (SfMNPV and SfGV) for the Control of the Fall Armyworm in Maize Crops

The fall armyworm, *Spodoptera frugiperda* (J.E. Smith) (Lepidoptera: Noctuidae) is a migratory pest endemic to the Americas that occurs from Southern Canada to Argentina [109]. It is a polyphagous insect that causes economic losses in several important crops, such as maize, sorghum, rice, cotton, and pastures [110]. Its control is based on the use of broad spectrum chemical insecticides or Bt transgenic crops, with the negative environmental effects and control failure due to development of resistance in the target pest [111,112,113,114,115].

In this context, the use of baculoviruses seems to be a promising alternative. Countries like Argentina [116,117], Brazil [118,119,120], Colombia [121], Honduras [122], Mexico [123,124,125,126], Peru [127], and Venezuela [128] have evaluated the effectiveness of geographical variants of SfMNPV in laboratory or field conditions, against local populations of S*. frugiperda*.

Naturally occurring SfMNPV has a wide genetic diversity [129]. Nine different genotypes were identified in the Nicaraguan isolate Sf-NIC [130,131]. From these nine genotypes, three were classified as defective (since they were not orally infective) and the rest of the genotypes were significantly less infective when compared individually against the complex wild type isolate. Interestingly, cell culture-co-occluded mixtures of complete and defective genotypes restored the pathogenicity of the virus to levels comparable with the complex wild type isolate [132,133,134].

This phenomenon, also found in a Colombian field isolate of SfMNPV consisting of at least ten distinct genotypes [135], is an interesting case of study of a heterogeneous population structured to optimize the viral fitness that poses challenges when a biopesticidal product is to be formulated. In a previous study, three native isolates of *Spodoptera frugiperda* multiple nucleopolyhedrovirus (SfMNPV) were characterized [136]. Moreover, biopesticidal formulations based on those viruses were obtained and field trials revealed that the isolates were effective in controlling the pest and keep the pest population below the economic damage threshold. The efficacy in controlling the pest were similar to that of chemical insecticides when treatment of plots was up to 22 days post emergence of the pest [137]. These studies allowed the development of a SfMNPV-based commercial formulation for the biological control of *S. frugiperda* by CORPOICA, which is in the process of registration [138].

In Brazil, an indigenous isolate of SfMNPV was used to control the insect in maize and was applied to 20,000 ha/year [47,139]. Owing to the high cost of SfMNPV production by EMBRAPA, this program has been discontinued [49]. Two major problems have limited the large-scale production of SfMNPV. First, the liquefaction of the integument as soon as the larvae die makes the process laborious and the final product expensive. Second, the cannibalistic behavior of the fall armyworm requires individual larval rearing, which is labor-intensive, increases the risk of contamination and raises the production costs [140].

Recently, an SfMNPV isolate that does not cause the liquefaction of the integument of larvae immediately after death was assayed in a two-step bioassay. The cannibalistic behaviour of *S. frugiperda* larvae and the number of OBs/larva produced was examined in an experimental design involving different size larvae fed on two food sources, maize (*Zea mays*) and castor bean (*Ricinus communis*) leaves inoculated with SfMNPV OBs. A decrease of cannibalistic behaviour and the highest number of OBs/larva was observed in larvae fed on castor bean leaves. By selecting the optimal larval instar and food for OB production, the amount of OB per larvae produced is higher and thus the number of larval equivalents (LE: number of larvae required to control 1 hectare) is lower [140].

The biopesticidal properties of several isolates of SfMNPV were also evaluated in Mexico. Different isolates varied significantly in their infectivity to its host, and only some had a potential to control the pest. Nevertheless, in field trials SfMNPV formulations have faced some shortcomings related to the low percentage of mortality achieved (less than 50%) and the insufficient environmental persistence of the OBs [141]. These limitations have led to the study of the stability of the formulations and the incorporation of UV protectors, phagostimulants, and the integration of SfMNPV in products containing azadiractin [142,143,144].

Recently, a Colombian *Spodoptera fugiperda* granulovirus was also characterized [24,145] and preliminary data suggest that, although this GV is very slow acting on its own, the addition of this baculovirus to SfNPV formulations may enhance NPVs biopesticidal performance, most likely due to enzymatic activities present in GV OBs [138].

### 2.5. Erinnyis ello GV for the Control of the Cassava Hornworm

Cassava (*Manihot esculenta*) is the basic source for dietary energy of 500 million people in tropical and subtropical areas of Africa, Asia, and Latin America [146]. The hornworm *Erinnyis ello* (Lepidoptera: Sphingidae) is an important pest which impacts cassava production in the Neotropics, causing complete plant defoliation, losses in bulk root production and reduced root quality. Together, these damages can cause yield losses of about 50%. It is also the most serious pest of the rubber tree (*Hevea brasiliensis*) in the New World. *E. ello* species tends to migrate in swarms, and fields can be completely defoliated with little warning when a swarm arrives and oviposits *en masse*. It is hypothesized that this migratory behavior is a possible defense against the large complex of natural enemies associated with *E. ello*, rendering natural biological control ineffective [147].

In 1973, the CIAT (International Center for Tropical Agriculture) found a virus infecting their *E. ello* colonies that caused the death of the larvae. This virus was later identified by G. Thomas at the University of Califirnia, in Berkeley [148] and classified as a granulovirus (*Erinnyis ello granulovirus*, ErelGV). This virus has been evaluated in the CIAT as a potential biological control agent. In field trials carried out in Brazil, damage caused by *E. ello* to cassava plantations ceased three days following the application of ErelGV [149].

Subsequently, a program for the production and distribution of the virus among farmers was implemented [149,150] in Southern Brazil by EMPASC (Empresa de Pesquisa Agropecuaria de Santa Catarina) in collaboration with CIAT. In addition, ErelGV was extensively used for hornworm control in Venezuela from the 1990s, being applied to more than 7000 hectares. In this case, the levels of control achieved were close to 100%, and the use of chemical pesticides was virtually eliminated [151].

The ErelGV genome was recently sequenced [22]. Apart from the importance of the molecular study of the virus, this information can be used for comparison of ErelGV isolates and for the assessment of the genetic stability of the isolates currently used in biopesticidal formulations.

*E. ello* is also an important pest for rubber trees and has been submitted for registration as a microbial control agent in Colombia by CORPOICA [138].

### 2.6. Other Baculoviruses Used as Biological Control Agents in Latin America

The Old World cotton bollworm *Helicoverpa armigera* (Lepidoptera: Noctuidae) was considered a quarantine pest in the Americas. Recently, its presence was detected in Brazil [152,153,154], Paraguay [155], and Argentina [156]. As mentioned in [157], it is possible that the introduction of this species occurred before the date of those reports, because the identification using external morphological characters of the larvae and adults of the *Helicoverpa*/*Heliothis* complex is difficult for a non-expert. Generally, before *H. armigera* was detected, the major damages in maize were caused by *H. zea*, while in soybean, chickpea and other legumes the incidence of *H. gelotopoeon* and *H. virescens* prevailed.

More recently, *H. armigera* has been reported to cause damages in cotton, soybean, corn, green beans, tomatoes, citrus and pastures, in Brazil [154]. To control this pest, emergency measures were established including the identification of the pest and approval of chemical and biological insecticides, as part of an intense effort carried out by research institutions such as EMBRAPA, universities, farmers associations and private enterprise. In this context, products based on baculovirus were imported and incorporated in the management of the pest (Table 1).

*Perigonia lusca* (Lepidoptera: Sphingidae) is an important pest of “yerba mate” also known as “Paraguay tea” in many European countries (*Ilex paraguariensis*) [158,159]. This pest was observed in Argentina, Paraguay [159], and Brazil [160]. The damage caused by *P. lusca* increased following the introduction of the monoculture in response to the increasing demands of yerba mate in South American countries [161]. A baculovirus (*Perigonia lusca* SNPV) was isolated from this species first in Argentina [162] and later in Brazil [161]. It has been successfully used by farmers as a crude preparation, in over 2500 ha of yerba mate plantations, at a dose equivalent to 15 infected last instar larvae per hectare [159,162]. *Perigonia lusca* SNPV genome has been recently sequenced [163]. Although no product is commercially available, the virus is currently used in IPM programs and for organic production of yerba mate in Argentina and Paraguay.

*Epinotia* (*=Crocidosema*) *aporema* (Lepidoptera: Tortricidae) is distributed from Southern USA to Argentina, Chile, and Uruguay. It produces variable losses in several leguminous crops including soybean. In Argentina, a betabaculovirus (*Epinotia aporema* GV, EpapGV) was isolated and characterized [164,165,166]. A formulation was assayed under controlled greenhouse conditions resulting in 80% larval mortality with a dose equivalent to 2 × 10^12^ OBs/hectare. Quality control procedures of the viral product were developed [167,168] and permission has been granted for experimental field studies.

During the 1990s Brazilian, Argentinean, and Uruguayan researchers carried out a cooperative project with the aim of evaluate baculovirus isolates that could be used to control *Chrysodeixis includens* (syn. *Pseudoplusia includes*) and *Rachiplusia nu* (Lepidoptera: Plusiinae) [169]. Nowadays, there is a renewed interest in the study on native isolates for the control of these pests, due to the increment in the insect populations that cause significant losses in soybean and other economically important crops in the region. In this regard, seven isolates of *Pseudoplusia includes* SNPV (PsinSNPV) collected from larvae present on cotton and soybean in Guatemala and Brazil, were characterized and evaluated [170,171]. The most virulent PsinSNPV-IE isolate was selected as candidate for the development of a biopesticide. Recently its genome was completely sequenced and analyzed [25].

In Argentina, two native isolates from *Rachiplusia nu* are being characterized and evaluated under laboratory conditions. One of them is an MNPV that could be considered a variant of *Autographa californica* MNPV with a different host range, while the other (designated RanuSNPV-SF92), seems to be a virus not yet described in the literature, which warrants further biological and molecular studies aimed at its characterization [172,173].

Among the biocontrol of forest pests, a baculovirus was isolated from larvae of *Condylorrhiza vestigialis* (Lepidoptera: Crambidae), a pest of poplar (Salicaceae: *Populus* sp.) plantations in Brazil [174]. A product based on the CoveMNPV was recently approved for commercial use in this country, under the brand name of Baculovirus Alamo.

## 3. Production Technology and Product Formulations

A formulation is the result of an active ingredient (such as baculovirus OBs) mixed with various components that improve the efficacy, stability and handling of the pesticide [175]. The basis for the formulation of baculovirus was set in the 1980s, making use of the formulation technologies previously developed for chemical pesticides [176]. In most cases, the product is formulated to optimize storage, and in the moment of use, it is suspended to obtain an applicable formulation. Infected larvae are dried by methods, such as dehydration [177], lyophilization [178], or by a humid air flow [179], to generate a powder. The application of lactose in the drying process improves the stability and infectivity of the virus [179]. To achieve the desired concentration, the powder is mixed with an inert carrier substance. The carrier must be cheap, not repellent for the larvae, and should keep the OBs well dispersed [180,181]. Silica and clays are commonly used carriers. Some of the components added to the formulation of application and their functions are listed in Table 2.

**Table 2:** Additives commonly used in baculovirus pesticide formulations.

Component	Function	References
Surfactants	Reduce the surface tension of the drops allowing the drops to be retained on the leaves. Facilitate the spread of drops that reach the leaves Act as emulsifier agent, allowing the oil to be mixed with water in the formulation.	[182]
Adherents	Increase adherence of the drops to the leaf surface.	[183]
Thickeners	Keep the formulation as a homogeneous mix.	[182,184,185]
Binders	Increase the tendency of the OB to adhere with the carrier.	[186,187]
Baits and phagostimulants	Attract the larvae to ingest the formulated pesticide. Attract natural enemies to the formulation.	[188,189]
UV protectors	Avoid the UV inactivation of the OB. Some of them also damage peritrophic membrane structure.	[190]

The liquid formulations are the most used when the biopesticide is applied to large areas. In this case, the OBs are suspended in water including an oily substance to avoid the evaporation of the droplets before they reach the surface of the plant [191].

One group of adjuvants of particular interest are collectively called optical brighteners, derived from stilbene compounds, such as Tinopal LPW, Blankophor BBH, Blankophor HRS, Blankophor P167, and Blankophor RKH. Since these compounds absorb UV radiation and emit light in the visible range, they were evaluated as UV protectors [192]. When the light protective activity of two optical brighteners (Tinopal CBS and Tinopal C1101) was evaluated, the results indicated SfMNPV OBs retained its biological activity against *S. frugiperda* larvae after 240 min of exposure to UV light [193]. In contrast, OBs without optical brighteners were completely inactivated after 15 min of exposure to UV light.

The addition of optical brighteners to *Spodoptera exigua* NPV not only increased the stability of the formulation, but also enhanced their pesticidal activity [194]. Some of the optical brighteners appear to exert their action by binding to chitin molecules, promoting degradation of the peritrophic membrane in the insect midgut and, thus, increasing the probability of infection of midgut epithelial cells [195,196].

Another optical brightener, Calcofluor M2R, was evaluated as enhancing factor in SfMNPV formulations [194]. When added to the formulation at a concentration of 0.1% p/v, it enhanced the pesticidal activity in 2.7, 6.5, and 61.6 times in second, third, and fourth instar *S. frugiperda* larvae, respectively. Moreover, the addition of Calcofluor M2R also lowered the CL50 of the biopesticide for third and fourth instar *S. frugiperda* larvae rendering it similar to that of second instar *S. frugiperda* larvae. These results indicate that optical brighteners, such as Calcofluor M2R, may be of help to control larvae from instars that are less susceptible to the virus.

There are other compounds that have demonstrated enhancing effects in the biopesticidal properties of baculovirus. For example, boric acid was found to reduce the median lethal time (LT50) of *A. gemmatalis* larvae infected with AgMNPV [197]. A more moderate effect was observed with SfMNPV [198]. Moreover, boric acid seemed to cause no effect on natural enemy populations at the concentrations used. It has been hypothesized that boric acid acts as a physiological stress factor, rendering the insect more susceptible to virus infection.

Microencapsulation has been evaluated as a strategy to maintain the components of the formulation in close contact. For example, *B. thuringiensis* and the NPV of *Heliotis* were encapsulated in starch granules [199]. Among the substances used to encapsulate are gelatin, pectin, chitin, calcium alginate and maize starch [200,201], although it has to be considered that the substance must not affect the viability of the virus, must not be alkaline and should dissolve easily in the insect midgut. For example, SfNPV was encapsulated in Eudragit-S100 microparticles (MPs), and the resulting particles were more resistant to UV-inactivation than OBs alone [202]. Microencapsulation seems promising for exploiting the activities of the components of a baculoviral formulation, but the possibilities that the technology offers have not been explored thoroughly.

Phagostimulants were studied as formulation components in the region as well [175,203]. In this respect, a granule recipe comprising of pregelatinized flour, starch, ground maize cob, maize oil, and water was identified that was evaluated for SfNPV. This formulation improved the efficacy and the stability of the pesticide in field [144].

In Latin America, baculovirus pesticides are produced *in vivo* either in-field or in insectaries. Production costs in cell culture are higher due to several reasons, including expensive culture media. To address this problem, studies on the metabolic and cytological aspects of *A. gemmatalis* cell line UFL-AG-286 and the effects of the culture medium and suspension culture on AgMNPV productivity were conducted in Argentina. These studies led to the adaptation of UFL-AG-286 to grow in agitated suspension cultures in spinner-flasks [204] and reduced the medium costs by replacing fetal bovine serum with low-price natural products [205,206]. With these adjustments, the cost of producing AgMNPV OB *in vitro* was reduced significantly, although there is still a long way ahead to achieve that of the production in larvae.

There have been many efforts to improve the economic efficiency of the production of baculovirus in cell cultures. Part of the improvement in cost efficiency comes from the advances in bioprocess technology and cell culture media formulations, a subject under continuous study [207,208,209]. These advances could, in future, be coupled with the development of transgenic cell lines with improved growth properties and OB productivity, a less explored possibility. Moreover, since the potential of lowering the costs is significant, production in cell cultures emerges as the key to achieving economic competitiveness with chemical pesticides. In addition, the viruses that exhibit a high speed of kill (either natural isolates or genetically modified viruses) can be mass-produced in cell culture due to the low production of OBs in insects that succumb to infection before a sufficiently large number of OBs can be produced. Alternatively, a tetracyclin-sensitive expression system has been developed allowing larvae to grow normal quantities of OBs [210].

## 4. Genetic Improvement of Baculovirus Biopesticides. Possibilities in Latin America

The baculoviruses have evolved to successfully infect their hosts, but they must overcome a series of obstacles to produce enough progeny and finally kill the insect [36]. The concept of genetically modifying the baculovirus to improve its endogenous insecticidal activity was developed in the United States during the 1980s [35,211,212,213]. The practical application of this concept was not long in coming: soon recombinant baculoviruses were developed and assayed in laboratory for their biopesticidal properties [214]. Since then, different strategies of genetic engineering have been explored to increase baculovirus speed of action. Several reviews covered the use, development, and ecology of genetically modified (GM) baculoviruses as biopesticides [215,216,217].

The first studies were conducted with a recombinant AcMNPV containing an insect-specific toxin gene [214]. Other strategies were based on the introduction of lepidopteran hormones that disrupt the normal physiology of the larvae [212,218,219,220]. A variant of this approach consisted in deleting or interrupting the viral ecdysteroid UDP-glycosyltransferase (*egt*) gene. The product of the viral *egt* gene prevents larval molting during infection, by inactivating ecdysone, thus increasing feeding activity of infected larvae and maximizing viral progeny [221,222]. The infection with an *egt* defective recombinant resulted in a moderate increase in the baculovirus speed of kill (about 20%–30%) and a more drastic reduction in food consumption and crop damage [222]. Other strategies are based on the insertion and expression of a group of baculovirus genes such as enhancins [223], cathepsins and chitinases [38] that damage the host peritrophic membrane resulting in an improved speed of colonization of the primary infected tissues compared to baculovirus lacking these genes.

Among the various genes evaluated to be inserted in the baculoviral genome, the most promising results were obtained with insect-specific toxins [214]. Insect predators and parasites use venoms to immobilize their prey in nature. Although arthropod venoms are in fact a mixture of toxins that may have a broad-spectrum activity against various organisms other than insects, it is possible to isolate toxin genes that target insects with high specificity.

In Latin America, genetic engineering of baculoviruses commonly used as biological control agents has been applied in studies aimed at improving their biopesticidal properties. Substantial progress has been made for the AgMNPV—*A. gemmatalis* system, starting with the development of recombinant occlusion-negative baculovirus expressing reporter genes [224,225] and the knockout of the *egt* gene [226]. More recently, a highly efficient system to produce AgMNPV recombinants was generated, which will be applied to test alternative genetic modifications aimed at improving biopesticidal parameters [227]. Additionally, an occlusion-negative AgMNPV was occluded in transgenic cell lines expressing AgMNPV polyhedrin [228]. The OBs produced can infect larvae orally, but no polyhedra can be formed in the larvae. The generation of OBs containing polyhedrin-negative baculovirus genomes has been previously proposed as a strategy to provide ecological containment [229], based on the observation that the oral infectivity and persistence of non-occluded virus in the environment is very low [230]. It is expected that this technology will allow a safer evaluation and application of genetically modified AgMNPV in field.

Recently, chitinase and cathepsin genes from *Choristonera fumiferana* DEF multiple nucleopolyhedrovirus (CfDEFNPV) were introduced in AgMNPV-2D genome (lacking these genes), and the recombinant showed a reduction of about 60% in the lethal concentration (LC_50_) for third instar *A. gemmatalis* larvae [231], and a moderate increase in the speed of kill.

A “bacmid” form of the SfMNPV genome that is able to replicate in *E. coli* was developed [232]. It is expected that this bacmid will facilitate the improvement of the biopesticidal properties of SfMNPV through genetic engineering.

Wild type AcMNPV is a component of the commercial biopesticide VPN ULTRA made in Guatemala by the private company Agrícola El Sol, and substitution with a GM AcMNPV should not pose an important technological problem. Many recombination systems are available and this facilitates the generation of GM virus. Moreover, tools are available to occlude polyhedrin-negative AcMNPV in complementing cell lines [228]. Many toxic genes have been evaluated in AcMNPV and field trials have been carried on with GM virus [229,230,233,234].

The legal regulations are very stringent regarding the incorporation of GM organisms in crops intended for human consumption; however, when enough data from controlled field trials become available, it is posssible to envisage a *niche* for pesticidal products based on recombinant baculovirus.

## 5. Conclusions and Perspectives

Over the previous two decades, the application of baculoviruses in Latin American countries has grown substantially and there are several reasons for this. First, the concern of some governments and the public about the effects caused by chemical pesticides generated support for the development of IPM programs and established incentives for organic production. Second, farmers faced with some of the drawbacks of chemical pesticides (e.g., resistance development and secondary pest resurgence), have adopted a greater openness towards the use of baculoviruses. Third, research advances have led to a larger inventory of diverse baculovirus species and to improvements in biopesticidal production technologies. Finally, the distribution of informative literature and activity of extensionists has had a positive influence on public perception and farmer’s acceptance of environmentally friendly microbial control agents. In parallel, regulations experienced changes in several countries of the region in order to favor the registration of biopesticides, use and commercial distribution.

However, the use of baculovirus-based pesticides has not reached its full potential. In this regard, it is important to consider that Latin America has one of the most diverse range of farming systems. Furthermore, in many farming systems the agricultural and socio-economic situations differs, and enterprises of large farmers (more than 500 ha, with prevalence of monoculture) contrast with small-holders, mainly family farmers. In general, pest management remains particularly dependent on broad-spectrum chemical insecticides, and the advance of the urbanization to rural areas increase the environmental problems. The regulatory frameworks vary between countries and even in different districts within each country. In such a way, the perspectives on the use of baculoviruses as microbial control agents can be analyzed in particular contexts.

For high-value products (such as apple, pear, or walnut), and especially in the commercial production for export, the demand of biopesticides has grown steadily due to restrictions on the traces of toxic chemicals in the final product. The market for CpGV-based pesticides and the demand of new products for the control of other lepidopteran pests in organic and conventional orchards is expanding. The perspectives for growing use of biopesticides in small and large horticultural farms are also very promising for the near future, and new products will be necessary.

A different scenario emerges in extensive agriculture, involving mainly soy and maize. The massive adoption of glyphosate-tolerant/Bt transgenic crops has lowered the demand of baculovirus products (such as AgMNPV), which became restricted to smaller extensions with non-transgenic crops and to organic production farms. It is possible that this situation will likely change due to the public concern about the cumulative effects of the pesticides in the environment, and to the recent emergence of resistance in certain insect populations.

Anyway, the scenario of a crop with a single economically important pest is unlikely to occur. In this context, there is a need to develop complex multispecies-baculovirus formulations that are able to control several pests simultaneously. In order to achieve this, it is necessary to increase the number of baculovirus species (or isolates) available and to find the most effective isolate(s) for each pest in each region. Moreover, the synergistic effect observed in the pesticidal activity among baculoviruses may encourage the development of mixed virus formulations. There are only eight indigenous baculoviruses completely sequenced [23,24,25,163,166,235,236,237], and this clearly indicates that the genetic diversity of baculoviruses has not been fully explored.

The research related to the scaling up of OBs production emerges as a key element to increase the competitiveness of baculovirus, especially for the protection of crops that are cultivated over large extensions of land. This is crucial in order to attract investments and produce baculovirus pesticides locally.

The results obtained in field trials of GM baculoviruses indicate that it is possible that the combination of the expression of insect-specific toxins, genes encoding enzymes that damage the insect midgut peritrophic membrane and genes aimed at interfering host physiology will enable to achieve a performance similar to the chemical insecticides. The combination of the mentioned elements has not been fully exploited so far. Furthermore, since there are diverse insect-specific toxins affecting different targets, it is likely that the simultaneous expression of two different toxins with different mechanisms of action will have an additive or synergistic effect on the performance of the biopesticide. Another factor that has not been fully explored is the timing and location of expression of the heterologous genes and their topology in the structure of the infectious virus. For example, chitinases, cathepsins and enhancins have an important effect in degrading the peritrophic membrane, before the virus enters the cell and starts its infective cycle. Thus, it is likely that the effects caused by these gene products are due to the presence of active proteins in the OBs. In this context, it is possible to envisage strategies to increase the effect of these proteins by routing them to the OB architecture. A similar case can be appreciated in GM baculoviruses expressing insect-specific toxic genes. The virus must enter the cell, the gene must be transcribed and the toxic protein must find its route to the target cell, limiting the speed of action of the transgene. If toxic gene products are incorporated as fusion proteins in OBs, they might act immediately after the virus enters the organism. Finally, approaches including gene silencing and the new DNA editing technologies have not yet been explored for the genetic improvement of baculoviral pesticide candidates [238,239].

In view of the complex population dynamics described for different virus-host systems, the impact of genetic modification on the performance of a particular baculovirus should be carefully evaluated by selecting the fittest genotype within the context of a formulation containing more than one genotype.

It is clear that the potential for the use of baculoviral pesticides in Latin America is enormous. The fulfilment of this potential will depend on the commitment of the governments in supporting biological control programs. This includes the continuous and consistent support of fundamental and applied research, the support to technology transfer from academic and agricultural research state agencies to private companies wishing to produce baculoviral pesticides, and education, training and actions to increase public awareness of the advantages of choosing biopesticidal products [240]. Finally, the cooperation between countries is extremely important in order to implement biological control programs, since there are many lepidopteran pests that are currently invading Mesoamerican and South American countries that are committed to ecologically sensitive manners of agricultural pest management and the preservation of biodiversity and the environment.

## References

[1] Wood R.J., Bishop J.A., Bishop J.A., Cook L.M. (1981). Insecticide resistance: Populations and evolution. Genetic Consequences of Man-Made Change.

[2] Whalon M.E., Mota-Sanchez D., Hollingworth R.M. (2008). Global Pesticide Resistance in Arthropods.

[3] Whalon M.E., Mota-Sanchez D., Hollingworth R.M., Duynslager L. Arthropod Pesticide Resistance Database. http://www.pesticideresistance.com.

[4] Fuller E., Elderd B.D., Dwyer G. (2012). Pathogen persistence in the environment and insect-baculovirus interactions: Disease-Density thresholds, epidemic burnout, and insect outbreaks. Am. Nat..

[5] Saxena H. (2008). Microbial Managment of Crop—Pest. J. Biopestic..

[6] Vasantharaj D.B. (2008). Biotechnological approaches in IPM and their impact on environment. J. Biopestic..

[7] Entwistle P.F., Evans H.F., Gilbert L.I., Kerkut G.A. (1985). Viral Control. Conprehensive Insect Fisiology. Biochemestry and Farmacology.

[8] Granados R.R., Federici B.A. (1986). The Biology of Baculoviruses.

[9] Moore N.F., King L.A., Possee R.D. (1987). Viruses of insects. Insect Sci. Appl..

[10] Tanada Y., Kaya H.K. (1993). Insect Pathology.

[11] Krieg A., Franz J.M., Groner A., Huber J., Miltenburger H.G. (1980). Safety of entomopathogenic viruses for control of insect pests. Environ. Conserve..

[12] Entwistle P.F. (1983). Viruses for insect pest control. Span.

[13] Cunningham J.C., Hunter-Fujita F.R., Entwistle P.F., Evans H.F., Crook N.E. (1998). Insect Viruses and Pest Management.

[14] Black B.C., Brennan L.A., Dierks L.A., Gard I.E., Miller L.K. (1997). Commercialization of baculoviral insecticides. The Baculoviruses.

[15] McWilliam A. (2007). Environmental Impact of Baculoviruses. http://www.fao.org/docs/eims/upload/agrotech/2003/R7299_FTR_anx3.pdf.

[16] Organization for Economic Co-operation and Development (OECD) Consensus Document on Information Used in the Assessment of Environmental Applications Involving Baculovirus. OECD Environment, Health and Safety Publications, Series on Harmonization of Regulatory Oversight in Biotechnology. http://www.rebeca-net.de/downloads/report/deliverable%2012.pdf.

[17] Rohrmann G.F. (2013). Baculovirus Molecular Biology: Third Edition (Internet). Bethesda (MD): National Center for Biotechnology Information (US). http://www.ncbi.nlm.nih.gov/books/NBK114593/.

[18] Herniou E.A., Arif B.M., Becnel J.J., Blissard G.W., Bonning B., Harrison R., Jehle J.A., Theilmann D.A., Vlak J.M., King A.M.Q., Adams M.J., Carstens E.B., Lefkowitz E.J. (2012). Baculoviridae. Virus Taxonomy: Classification and Nomenclature of Viruses: Ninth Report of the International Committee on Taxonomy of Viruses.

[19] Bonning B.C., Kostas I., Lawrence G., Sarjeet G. (2005). Baculoviruses: Biology, biochemistry, and molecular biology. Comprehensive Molecular Insect Science.

[20] Jehle J.A., Lange M., Wang H., Hu Z., Wang Y., Hauschild R. (2006). Molecular identification and phylogenetic analysis of baculoviruses from Lepidoptera. Virology.

[21] Jehle J.A., Blissard G.W., Bonning B.C., Cory J.S., Herniou E.A., Rohrmann G.F., Theilmann D.A., Thiem S.M., Vlak J.M. (2006). On the classification and nomenclature of baculoviruses: A proposal for revision. Arch. Virol..

[22] Ardisson-Araújo D.M., de Melo F.L., Andrade M.S., Sihler W., Báo S.N., Ribeiro B.M., de Souza M.L. (2014). Genome sequence of *Erinnyis ello granulovirus* (ErelGV), a natural cassava hornworm pesticide and the first sequenced sphingid-infecting betabaculovirus. BMC Genomics.

[23] Wennmann J.T., Gueli Alletti G., Jehle J.A. (2014). The genome sequence of *Agrotis segetum* nucleopolyhedrovirus B (AgseNPV-B) reveals a new baculovirus species within the Agrotis baculovirus complex. Virus Genes.

[24] Cuartas P.E., Barrera G.P., Belaich M.N., Barreto E., Ghiringhelli P.D., Villamizar L.F. (2015). The Complete Sequence of the First *Spodoptera frugiperda Betabaculovirus* Genome: A Natural Multiple Recombinant Virus. Viruses.

[25] Craveiro S.R., Inglis P.W., Togawa R.C., Grynberg P., Melo F.L., Ribeiro M.A., Ribeiro B.M., Báo S.N., Castro M.L.B. (2015). The genome sequence of *Pseudoplusia includens* single nucleopolyhedrovirus and an analysis of p26 gene evolution in the baculoviruses. BMC Genomics.

[26] Adams J.R., McClintock J.T., Adams J.R., Bonami J.R. (1991). Baculoviridae, nuclear polyhedrosis viruses Part 1: Nuclear polyhedrosis viruses of insects. Atlas of Invertebrate Viruses.

[27] Haase S., Ferrelli M.L., Pidre M.L., Romanowski V. Genetic Engineering of Baculoviruses, Current Issues in Molecular Virology—Viral Genetics and Biotechnological Applications. http://www.intechopen.com/books/current-issues-in-molecular-virology-viral-genetics-and-biotechnological-applications/genetic-engineering-of-baculoviruses.

[28] Hegedus D., Erlandson M., Gillott C., Toprak U. (2009). New insights into peritrophic matrix synthesis, architecture, and function. Annu. Rev. Entomol..

[29] Barbehenn R.V., Martin M.M. (1995). Peritrophic envelope permeability in herbivorous insects. J. Insect Physiol..

[30] Wang P., Granados R.R. (1997). An intestinal mucin is the target substrate for a baculovirus enhancin. Proc. Natl. Acad. Sci. USA.

[31] Hashimoto Y., Corsaro B.G., Granados R.R. (1991). Location and nucleotide sequence of the gene encoding the viral enhancing factor of the *Trichoplusia ni* granulosis virus. J. Gen. Virol..

[32] Slavicek J.M., Popham H.J. (2005). The *Lymantria dispar* nucleopolyhedrovirus enhancins are components of occlusion-derived virus. J. Virol..

[33] Derksen A.C.G., Granados R.R. (1988). Alteration of a lepidopteran peritrophic membrane by baculoviruses and enhancement of viral infectivity. Virology.

[34] Tanada Y., Hess R.T., Omi E.M. (1975). Invasion of a nuclear polyhedrosis virus in midgut of the armyworm, *Pseudaletia unipuncta*, and the enhancement of a synergistic enzyme. J. Invertebr. Pathol..

[35] Miller L.K., Lingg A.J., Bulla L.A. (1983). Bacterial, viral, and fungal insecticides. Science.

[36] Passarelli A.L. (2011). Barriers to success: How baculovirus establish efficient systemic infections. J. Virol..

[37] Van Oers M.M., Flipsen J.T., Reusken C.B., Vlak J.M. (1994). Specificity of baculovirus p10 functions. Virology.

[38] Hawtin R.E., Zarkowska T., Arnold K., Thomas C.J., Gooday G.W., King L.A., Kuzio J.A., Possee R.D. (1997). Liquefaction of *Autographa californica* Nucleopolyhedrovirus-infected insects is dependent on the integrity of virus-encoded chitinase and cathepsin genes. Virology.

[39] Vasconcelos S.D. (1996). Alternative Routes for the Horizontal Transmission of a Nucleopolyhedrovirus. J. Invertebr. Pathol..

[40] Fuxa J.R., Richter A.R., Ameen A.O., Hammock B.D. (2002). Vertical transmission of TnSNPV, TnCPV, AcMNPV, and possibly recombinant NPV in *Trichoplusia ni*. J. Invertebr. Pathol..

[41] Bird J.T. (1953). The use of a virus disease in the biological control of the European pine sawfly, *Neodiprion sertifer* (Geoffr). Can. Entomol..

[42] Bird J.T., Elgee D.E. (1957). A virus disease and introduced parasites as factors controlling the European spruce sawfly, *Diprion hercyniae* Htd. in central New Brunswick. Can. Entomol..

[43] Fuxa J.R. (2004). Ecology of insect nucleopolyhedroviruses. Agric. Ecosyst. Environ..

[44] Kogan M., Turnipseed S.G., Shepard M., Oliveira E.B., Borgo A. (1977). Pilot insect pest management for soybean in southern Brazil. J. Econ. Entomol..

[45] Allen G.E., Knell J.D. (1977). A nuclear polyhedrosis virus of *Anticarsia gemmatalis* I: Ultrastructure, replication, and pathogenicity. Fla. Entomol..

[46] Carner G.R., Turnipseed S.G. (1977). Potential of a nuclearpolyhedrosis virus for the control of the velvetbean caterpillar in soybean. J. Econ. Entomol..

[47] Moscardi F. (1999). Assessment of the application of baculoviruses for the control of Lepidoptera. Ann. Rev. Entomol..

[48] Moscardi F., Vincent C., Goethel M.S., Lazarovits G. (2007). A Nucleopolyhedrovirus for control of the velvetbean caterpillar in Brazilian Soybeans. Biological Control: A Global Perspective.

[49] Moscardi F., de Souza Lobo M., de Castro Batista M.E., Moscardi L.M., Szewczyk B., Ahmad I., Ahmad F., Pichtel P. (2011). Baculovirus pesticides: Present state and future perspectives. Microbes and Microbial Technology.

[50] Moscardi F. (1989). Use of viruses for pest control in Brazil: The case of the nuclear polyhedrosis virus of the soybean caterpillar, *Anticarsia gemmatalis*. Mem. Inst. Oswaldo Cruz.

[51] Szewczyk B., Rabalski L., Krol E., Sihler W., Lobo de Souza M. (2009). Baculovirus biopesticides—A safe alternative to chemical protection of plants. J. Biopestic..

[52] Sosa-Gómez D.R., Moscardi F., Santos B., Alves L.F.A., Alves S.B., Alves S.A., Lopes R.B. (2008). Produçao e uso de vírus para o controle de pragas na América Latina. Controle Microbiano de Pragas na América Latina: Abanicos e Desafíos.

[53] Moscardi F., Sosa-Gómez D.R., Lacey L.A., Kaya H.K. (2000). Microbial control of insect pests of soybeans. Field Manual of Techniques in Invertebrate Pathology: Application and Evaluation of Pathogens for Control of Insects and Other Invertebrate Pests.

[54] Santos B. (2003). Avanços na Produção Massal de Lagartas de *Anticarsia gemmatalis* Hübner 1818 (Lepidoptera: Noctuidae) Infectadas Com o Seu Vírus de Poliedrose Nuclear, em Laboratório e do Bioinseticida à Base Desse Vírus. Ph.D. Thesis.

[55] Corrêa-Ferreira B.S., Alexandre T.M., Pellizzaro E.C., Moscardi F., Bueno A.F. (2010). Práticas de manejo de pragas utilizadas na soja e seu impacto sobre a cultura.

[56] Bueno R.C.O.F., Parra J.R.P., Bueno A.F., Moscardi F., Oliveira J.R.G., Camillo M.F. (2007). Sem barreira. Revista Cultivar.

[57] Sosa-Gómez D.R. (2014). Personal Communication.

[58] Kokubu H. Brief overview of microorganisms used against agricultural insect pests. http://e-cucba.cucba.udg.mx/index.php/e-Cucba/article/view/10/pdf_brief.

[59] Williams T., Arredondo-Bernal H.C., Rodríguez-del-Bosque L.A. (2013). Biological Pest Control in Mexico. Annu. Rev. Entomol..

[60] Tanada Y. (1964). A granulosis virus of the codling moth, *Carpocapsa pomonella* (Linnaeus) (Olethreutidae, Lepidoptera). J. Insect Pathol..

[61] Falcon L.A., Kane W.R., Bethel R.S. (1968). Preliminary evaluation of a granulosis virus for control of the codling moth. J. Econ. Entomol..

[62] Lacey L., Thomson D., Vincent C., Arthurs S.P. (2008). Codling moth granulovirus: A comprehensive review. Biocontrol Sci. Technol..

[63] Beas-Catena A., Sánchez-Mirón A., García-Camacho F., Contreras-Gómez A., Molina-Grima E. (2014). Baculovirus biopesticides: An overview. J. Anim. Plant Sci..

[64] Quintana G., Alvarado L. (2004). Carpovirus Plus: Primer insecticida biológico para el control de *Cydia pomonella* en montes comerciales de pera, manzana y nogal. AgroInnova—La Innovación Tecnológica para Mejorar la Competitividad.

[65] Quintana G. (2009). Control biológico de *Cydia pomonella*: Virus de la granulosis de *Cydia pomonella*: Una alternativa segura y eficaz de control. Rev. Redagrícola.

[66] Quintana G. (2013). Uso del virus de la granulosis (CpGV) para el control de carpocapsa (*Cydia pomonella* L.) en Argentina. Resúmenes TAMIBIO 2013, DiMAyA. Asoc. Argent. Microbiol..

[67] Quintana G., Cólica J.J., del Fernández Górgola M.C., Rivero C., Pérez O., Luna Mercado L. (2007). Control de carpocapsa (*Cydia pomonella* L.) con un producto en base al virus de la granulosis (CpGV), en cultivos de nogal en Catamarca. Rev. CIZAS.

[68] Cólica J., Quintana G., del Fernández Górgola M.C., la Rossa R. (2008). Evaluación preliminar de una formulación atracticida para el control de *Cydia pomonella* (L) en montes de nogal en Catamarca, Argentina. Revista del CIZAS. Facultad de Ciencias Agrarias, Universidad Nacional de Catamarca (UNCa). Rev. Cient. CIZAS.

[69] Sauphanor B., Berling M., Toubon J.K., Reyes M., Delnatte J., Allemoz P. (2006). Carpocapse des pommes: Cas de resistance au virus de la granulose en vergers biologiques. Phytoma Def.Veg..

[70] Asser K.S., Fritsch E., Undorf S.K., Kienzle J., Eberle K.E., Gund N.A., Reineke A., Zebitz C.P., Heckel D.G., Huber J. (2007). Rapid emergence of baculovirus resistance in codling moth due to dominant, sex-linked inheritance. Science.

[71] Eberle K.E., Jehle J.A. (2006). Field resistance of codling moth against *Cydia pomonella* granulovirus (CpGV) is autosomal and incompletely dominant inherited. J. Invertebr. Pathol..

[72] Schmitt A., Bisutti I.L., Ladurner E., Benuzzi M., Sauphanor B., Kienzle J., Zingg D., Undorf-Spahn K., Fritsch E., Huber J. (2013). The occurrence and distribution of resistance of codling moth to *Cydia pomonella* granulovirus in Europe. J. Appl. Entomol..

[73] Zichová T., Stará J., Kundu J., Eberle K.E., Jehle J.A. (2013). Resistance to *Cydia pomonella* granulovirus follows a geographically widely distributed inheritance type within Europe. BioControl.

[74] Rezapanah M., Shojai-Estabragh S., Huber J., Jehle J.A. (2008). Molecular and biological characterization of new isolates of *Cydia pomonella* granulovirus from Iran. J. Pest Sci..

[75] Eberle K.E., Asser-Kaiser S., Sayed S.M., Nguyen H.T., Jehle J.A. (2008). Overcoming the resistance of codling moth against conventional *Cydia pomonella* granulovirus (CpGV-M) by a new isolate CpGV-I12. J. Invertebr. Pathol..

[76] Berling M., Blachere-Lopez C., Soubabere O., Lery X., Bonhomme A., Sauphanor B., Lopez-Ferber M. (2009). *Cydia pomonella* granulovirus Genotypes Overcome Virus Resistance in the Codling Moth and Improve Virus Efficiency by Selection against Resistant Hosts. Appl. Environm. Microbiol..

[77] Arneodo J.D., de Anna J., Salvador R., Farinon M., Quintana G., Sciocco-Cap A. (2015). Prospection and molecular analysis of CpGV isolates infecting *Cydia pomonella* at different geographical locations in Argentina. Ann. Appl. Biol..

[78] Quintana G. (2014). Personal Communication.

[79] Ríos-Velasco C., Sánchez-Valdez V.M., Gallegos-Morales G., Cambero-Campos O.J. (2012). Evaluación en campo del granulovirus CpGV sobre *Cydia pomonella* L. (Lepidoptera: Tortricidae) CpGV field evaluation on *Cydia pomonella* L. (Lepidoptera: Tortricidae). Rev. Mex. Cienc. Agríc..

[80] International Potato Center (CIP). http://cipotato.org.

[81] Barragán A.R., Onore G., Zeddam J.L. (2005). Identificación, biología, y comportamiento de las polillas de la papa en el Ecuador.

[82] Rondon S.I. (2010). The potato tuber moth: A literature review of its biology, ecology and control. Am. J. Potato Res..

[83] Raman K.V., Booth R.H., Palacios M. (1987). Control of potato tuber moth *Phthorimaea operculella* (Zeller) in rustic potato stores. Trop. Sci..

[84] Kroschel J., Sporleder M., Tonnang H.E.Z., Juarez H., Carhuapoma P., Gonzales J.C., Simon R. (2013). Predicting climate-change-caused changes in global temperature on potato tuber moth *Phthorimaea operculell*a (Zeller) distribution and abundance using phenology modeling and GIS mapping. Agric. For. Meteorol..

[85] Reed E.M., Springett B.P. (1971). Large-scale field testing of a granulosis virus for the control of the potato moth (*Phthorimaea operculella* (Zell.) (Lep., Gelechiidae)). Bull. Entomol. Res..

[86] Matthiessen J.N., Christian R.L., Grace T.D.C., Filshie B.K. (1978). Large-scale field propagation and the purification of the granulosis virus of the potato moth, *Phthorimaea operculella* (Zeller) (Lepidoptera: Gelechiidae). Bull. Entomol. Res..

[87] Alcázar J., Raman K.V., Salas R. (1991). Un virus como agente de control de la polilla de la papa *Phthorimaea operculella*. Rev. Peru. Entomol..

[88] Kroschel J., Kaack H.J., Fritsch E., Huber J. (1996). Biological control of the potato tuber moth (*Phthorimaea operculella* Zeller) in the Republic of Yemen using granulosis virus: Propagation and effectiveness of the virus in field trials. Biocontrol Sci. Technol..

[89] Zeddam J.L., Pollet A., Mangoendiharjo S., Ramadhan T.H., Lopez-Ferber M. (1999). Occurrence and virulence of a granulosis virus in *Phthorimaea operculella* (Lep. Gelechiidae) populations in Indonesia. J. Invertebr. Pathol..

[90] Laarif A., Fattouch S., Essid W., Marzouki N., Salah H.B., Hammouda M.H.B. (2003). Epidemiological survey of *Phthorimaea operculella* granulosis virus in Tunisia. EPPO Bull..

[91] Lacey L.A., Kroschel J., Arthurs S.P., de la Rosa F. (2010). Control microbiano de la palomilla de la papa *Phthorimaea operculella* (Lepidoptera: Gelechiidae). Rev. Colomb. Entomol..

[92] Raman K.V., Alcazar J., Valdez A. (1992). Biological Control of the Potato Tuber Moth Using Phthorimaea Baculovirus.

[93] Barea O., Bejarano C., Calderón R., Crespo L., Franco J., Herbas J., Lino V., Martínez E., Ramos J. (2002). http://www.asocam.org/biblioteca/files/original/35b23c7f7ae28eae20b35f797ad89b5b.pdf.

[94] Suquillo J., Rodríguez P., Gallegos P., Orbe K., Zeddam J.L. (2012). Manual Para la Elaboración del Bioinsecti*cida Bacu-Turin a Travé*s d*e Premezclas Concentrada*s Para el Control de las Polillas de la Papa: *Tecia Solanivora, Phthorimaea Operculella y Symmetrischema Tangolia*.

[95] Briese D.T., Mende H.A. (1981). Differences in susceptibility to a granulosis virus between field populations of the potato moth, *Phthorimaea Operculella* (Zeller) (Lepidoptera: Gelechiidae). Bull. Entomol. Res..

[96] Vickers J.M., Cory J.S., Entwistle P.F. (1991). DNA characterization of eight geographic isolates of granulosis virus from the potato tuber moth (*Phthorimaea operculella*) (lepidoptera, gelechiidae). J. Invertebr. Pathol..

[97] Sporleder M., Kroschel J. (2003). The granulovirus of the potato tuber moth *Phthorimaea operculella* (Zeller): Characterization and prospects for effective mass production and pest control. Advances in Crop Research Volume 3.

[98] Zeddam J.L., Léry L., Gómez-Bonilla Y., Espinel-Correal C., Páez D., Rebaudo F., López-Ferber M. (2013). Responses of different geographic populations of two potato tuber moth species to genetic variants of *Phthorimaea operculella* granulovirus. Entomol. Exp. Appl..

[99] Ángeles I., Alcázar J. (1995). Susceptibilidad de la polilla *Scrobipalpuloides absoluta* al virus de la granulosis de *Phthorimaea operculella* (PoVG). Rev. Peru. Entomol..

[100] Niño L., Notz A. (2000). Patogenicidad de un virus granulosis de la polilla de la papa *Tecia solanivora* (Povolny 1973) (Lepidoptera: Gelechiidae) en el estado de Mérida, Venezuela. Bol. Entomol. Venez..

[101] Zeddam J.L., Vásquez M., Vargas Z., Lagnaoui A. (2003). Producción viral y tasas de aplicación del granulovirus usado para el control biológico de las polillas de la papa *Phthorimaea operculella*
*y Tecia solanivora* (Lepidoptera: Gelechiidae). Plagas.

[102] Lery X., Villamizar L., Espinel C., Zeddam J.L., Cotes A.M., López-Ferber M. (2008). Analysis of several Colombian *Phthorimaea operculella* granuloviruses isolated from *Tecia solanivora*: Detection of a new variable region in the PhopGV genome. IOBC/WPRS Bull..

[103] Cuartas P.O., Villamizar L., Espinel C.C., Cotes A.M. (2009). Infección de granulovirus nativos sobre *Tecia solanivora* y *Phthorimaea operculella* (Lepidoptera: Gelechiidae). Rev. Colomb. Entomol..

[104] Espinel-Correa C., Lery X., Villamizar L., Gómez J., Zeddam J.L., Cotes A.M., López-Ferber M. (2010). Genetic and biological analysis of Colombian *Phthorimaea operculella* Granulovirus isolated from *Tecia solanivora* (Lepidoptera: Gelechiidae). Appl. Environ. Microbiol..

[105] Espinel-Correa C., López-Ferber M., Zeddam J.L., Villamizar L., Gómez J., Cotes A.M., Léry X. (2012). Experimental mixtures of *Phthorimaea operculella* granulovirus isolates provide high biological efficacy on both *Phthorimaea operculella* and *Tecia solanivora* (Lepidoptera: Gelechiidae). J. Invertebr. Pathol..

[106] Gómez-Bonilla Y., López-Ferber M., Caballero P., Léry X., Muñoz D. (2011). Characterization of a Costa Rican granulovirus strain highly pathogenic against its indigenous hosts, *Phthorimaea operculella* and *Tecia solanivora*. Entomol. Exp. Appl..

[107] Carpio C., Olivier D., Dupas S., Léry X., Lopez-Ferber M., Orbe K., Páez D., Rebaudo F., Santillan A., Yangari B. (2013). Development of a viral biopesticide for the control of the Guatemala potato tuber moth *Tecia solanivora*. J. Invertebr. Pathol..

[108] Moura Mascarin G., Batista Alves S., Rampelotti-Ferreira F.T., Ragassi Urbano M., Borges Demétrio C.G., Delalibera I. (2010). Potential of a granulovirus isolate to control *Phthorimaea operculella* (Lepidoptera: Gelechiidae). BioControl.

[109] Johnson S.J. (1987). Migration and the life history strategy of the fall armyworm, *Spodoptera frugiperda*, in the Western hemisphere. Insect Sci. Appl..

[110] Clark P.L., Molina-Ochoa J., Martinelli S., Skoda S.R., Isenhour D.I., Lee D.J., Krumm J.T., Foster J.E. (2007). Population variation of the fall armyworm, *Spodoptera frugiperda*, in western hemisphere. J. Insect Sci..

[111] Yu S.J. (1992). Detection and biochemical characterization of insecticide resistance in fall armyworm (Lepidoptera: Noctuidae). J. Econ. Entomol..

[112] Pacheco-Covarrubias J.J. (1993). Monitoring insecticide resistance in *Spodoptera frugiperda* populations from the Yaqui Valley, Son., Mexico. Resist. Pest Manag. Newsl..

[113] Storer N.P., Babcock J.M., Schlenz M., Meade T., Thompson G.D., Bing J.W., Huckaba R.M. (2010). Discovery and characterization of field resistance to Bt maize: *Spodoptera frugiperda* (Lepidoptera: Noctuidae) in Puerto Rico. J. Econ. Entomol..

[114] Ríos-Díez J.D., Saldamando-Benjumea C.I. (2011). Susceptibility of *Spodoptera frugiperda* (Lepidoptera: Noctuidae) strains from central Colombia to two insecticides, Methomyl and Lambda-Cyhalothrin: A study of the genetic basis of resistance. J. Econ. Entomol..

[115] León-García I., Rodríguez-Leyva E., Ortega-Arenas L.D., Solís-Aguilar J.E. (2012). Susceptibilidad de *Spodoptera frugiperda* (J.E. Smith) (Lepidoptera: Noctuidae) a insecticidas asociada a césped en Quintana Roo, México. Agrociencia.

[116] Berretta M.F., Rios M.L., Sciocco de Cap A. (1998). Characterization of a nuclear polyhedrosis virus of *Spodoptera frugiperda* from Argentina. J. Invertebr. Pathol..

[117] Yasem de Romero M.G., Romero E., Sosa Gómez D., Willink E. (2009). Evaluación de aislamientos de baculovirus para el control de *Spodoptera frugiperda* (Smith, 1797) Lep.: Noctuidae, plaga clave del maíz en el noroeste argentino. Rev. Ind. Agríc. Tucumán.

[118] Valicente F.H., Peixoto M.J.V.V.D., Paiva E., Kitajima E.W. (1989). Identificação e purificação de um vírus da poliedrose nuclear da lagarta *Spodoptera frugiperda* (J.E. Smith, 1797) (Lepidoptera: Noctuidae). An. Soc. Entomol. Bras..

[119] Valicente F.H., da Costa E.F. (1995). Controle da lagarta do cartucho *Spodoptera frugiperda* (J.E. Smith) com baculovirus spodoptera, aplicado via água de irrigação. An. Soc. Entomol. Bras..

[120] Arce Gómez S., Moscardi F., Sosa Gómez D.R. (1999). Susceptibilidade de *Spodoptera frugiperda* a isolados geograficos de um virus de poliedrose nuclear. Pesqui. Agropecu. Bras..

[121] Gómez Valderrama J.A., Guevara Agudelo E.J., Barrera Cubillos G.P., Cotes Prado A.M., Villamizar Rivero L.F. (2010). Aislamiento, identificación y caracterización de nucleopoliedrovirus nativos de *Spodoptera frugiperda* en Colombia. Rev. Fac. Nal. Agron. Medellín.

[122] Williams T., Goulson D., Caballero P., Cisneros J., Martínez A.M., Chapman J.W., Roman D.X., Cave R. (1999). Evaluation of a baculovirus bioinsecticide for small-scale maize growers in Latin America. Biol. Control.

[123] Martínez A.M., Goulson D., Chapman J.W., Caballero P., Cave R.D., Williams T. (2000). Is it feasible to use optical brightener technology with a baculovirus insecticide for resource-poor maize farmers in Mesoamerica?. Biol. Control.

[124] Rangel Núñez J.C., Vázquez Ramírez M.F., del Rincón Castro M.C. (2014). Caracterización biológica y molecular de cepas exóticas de Baculovirus SfNPV, con actividad bioinsecticida hacia una población mexicana del gusano cogollero del maíz *Spodoptera frugiperda* (Lepidóptera: Noctuidae). Interciencia.

[125] Ríos-Velasco C., Gallegos-Morales G., Berlanga-Reyes D., Cambero-Campos J., Romo-Chacón A. (2012). Mortality and Production of Occlusion Bodies in Spodoptera frugiperda Larvae (Lepidoptera: Noctuidae) Treated with Nucleopolyhedrovirus. Fla. Entomol..

[126] García-Gutiérrez C., Escobedo-Bonilla C.M., López M.A. (2013). Infectivity of a Sinaloa Native Isolate of Multicapsid Nuclear Polyhedrosis Virus (SfMNPV) against Fall Armyworm, *Spodoptera frugiperda* (Lepidoptera: Noctuidae). Southwest. Entomol..

[127] Vásquez J., Zeddam J.L., Tresierra A.A. (2002). Control biológico del “cogollero del maíz” *Spodoptera*
*frugiperda*, (Lepidoptera: Noctuidae) con el Baculovirus SfVPN, en Iquitos-Perú. Folia Amazon..

[128] Agudelo F., Romano M., Wassink H., Cuello de Uzcategui R. (1983). Una poliedrosis de *Spodoptera frugiperda* en Venezuela. Turrialba.

[129] Simón O., Williams T., López-Ferber M., Caballero P. (2004). Genetic structure of a *Spodoptera frugiperda nucleopolyhedrovirus* population: High prevalence of deletion genotypes. Appl. Environ. Microbiol..

[130] Simón O., Chevenet F., Williams T., Caballero P., López-Ferber M. (2005). Physical and partial genetic map of *Spodoptera frugiperda* nucleopolyhedrovirus (SfMNPV) genome. Virus Genes.

[131] Simón O., Williams T., Cerutti M., Caballero P., López-Ferber M. (2013). Expression of a Peroral Infection Factor Determines Pathogenicity and Population Structure in an Insect Virus. PLoS ONE.

[132] López-Ferber M., Simón O., Williams T., Caballero P. (2003). Defective or effective? Mutualistic interactions between virus genotypes. Proc. R. Soc. B Biol. Sci..

[133] Clavijo G., Williams T., Simón O., Muñoz D., Cerutti M., López-Ferber M., Caballero P. (2009). Mixtures of complete and *pif1*- and *pif2*-deficient genotypes are required for increased potency of an insect nucleopolyhedrovirus. Virology.

[134] Clavijo G., Williams T., Muñoz D., Caballero P., López-Ferber M. (2010). Mixed genotype transmission bodies and virions contribute to the maintenance of diversity in an insect virus. Proc. R. Soc. B.

[135] Barrera G., Williams T., Villamizar L., Caballero P., Simón O. (2013). Deletion Genotypes Reduce Occlusion Body Potency but Increase Occlusion Body Production in a Colombian *Spodoptera frugiperda* Nucleopolyhedrovirus Population. PLoS ONE.

[136] Villamizar L., Guevara J., Espinel C., Gómez M., Gómez J., Cuartas Paola., Barrera G., Cruz M., Santos A., Uribe L. (2012). Desarrollo de un Bioplaguicida a Base de Nucleopoliedrovirus Para el Control del Gusano Cogollero del Maíz, Spodoptera Frugiperda.

[137] Gómez J., Guevara J., Cuartas P., Espinel C., Villamizar L. (2013). Microencapsulated Spodoptera frugiperda nucleopolyhedrovirus: Insecticidal activity and effect on arthropod populations in maize. Biocontrol Sci. Technol..

[138] Villamizar L. (2015). Personal Communication.

[139] Valicente F.H., Cruz I. (1991). Controle Biológico da Lagarta do Cartucho, Spodoptera Frugiperda, Com o Baculovirus.

[140] Valicente F.H., Tuelher E.S., Pena R.C., Andreazza R., Guimarães M.R.F. (2013). Cannibalism and virus production in *Spodoptera frugiperda* (J.E. Smith) (Lepidoptera: Noctuidae) larvae fed with two leaf substrates inoculated with baculovirus spodoptera. Neotrop. Entomol..

[141] Martínez A.M., Pineda S., Figueroa J.I., Chavarrieta J.M., Williams T. (2012). Los baculovirus como bioinsecticidas: Evaluación de un nucleopoliedrovirus para el combate de *Spodoptera frugiperda* (Lepidoptera: Noctuidae) en México y Honduras. Cienc. Nicolaita.

[142] Zamora-Avilés N., Alonso-Vargas J., Pineda S., Isaac-Figueroa J., Lobit P., Martínez-Castillo A.M. (2013). Effects of a nucleopolyhedrovirus in mixtures with azadirachtin *on Spodoptera frugiperda* (JE Smith) (Lepidoptera: Noctuidae) larvae and viral occlusion body production. Biocontrol Sci. Technol..

[143] Pineda S., Pérez-Robledo C.A., Hernández R.E., de la Rosa J.F., Chavarrieta J.M., Martínez A.M. (2014). Combined and individual effects of a nucleopolyhedrovirus and azadirachtin on the mortality and maize-leaf consumption of *Spodoptera frugiperda*. Phytoparasitica.

[144] Castillejos V., Trujillo J., Ortega L.D., Santizo J.A., Cisneros J., Penagos D.I., Valle J., Williams T. (2002). Granular phagostimulant nucleopolyhedrovirus formulations for control of S*podoptera frugiperda* in maize. Biol. Control.

[145] Cuartas P.O., Barrera G., Barreto E., Villamizar L. (2014). Characterization of a Colombian granulovirus (Baculoviridae: Betabaculovirus) isolated from *Spodoptera frugiperda* (Lepidoptera: Noctuidae) larvae. Biocontrol Sci. Technol..

[146] El-Sharkawy M.A. (2004). Cassava biology and physiology. Plant Mol. Biol..

[147] Bellotti A.C., Arias B., Guzman O.L. (1992). Biological control of the cassava hornworm *Erinnyis ello* (Lepidoptera: Sphingidae). Fla. Entomol..

[148] Bellotti A.C., Arias B., Reyes J.A., Ospina B., Ceballos H. (2002). Manejo de plagas de la yuca. La Yuca en el Tercer Milenio. Sistemas Modernos de Producción, Procesamiento, Utilización y Comercialización.

[149] Schmitt A.T. (1988). Using Baculovirus erinnyis in the biological control of cassava hornworm. Cassava Newsl..

[150] Bellotti A.C., Arias B., Brekelbaum T., Bellotti A., Lozano J.C. (1978). Biology, ecology and biological control of the cassava hornworm (*Erinnyis ello*). Cassava Protection Workshop.

[151] Laberry R. (1977). La Aplicación de un Programa MIP en Producción Industrial de Yuca. En: Memorias del Congreso Biodiversidad y Micorrizas.

[152] Czepak C., Cordeiro Albernaz K., Vivan L.M., GuiMarães H.O., Carvalhais T. (2013). Primeiro registro de ocorrência de *Helicoverpa armigera* (Hübner) (Lepidoptera: Noctuidae) no Brasil. Pesqui. Agropecu. Trop., Goiâ..

[153] Specht A., Sosa-Gómez D.R., Vieira de Paula-Moraes S., Cavaguchi Yano S.A. (2013). Identificação morfológica e molecular de *Helicoverpa armigera* (Lepidoptera: Noctuidae) e ampliação de seu registro de ocorrência no Brasil. Pesqui. Agropecu. Bras. (Brasília).

[154] Tay W.T., Soria M.F., Walsh T., Thomazoni D., Silvie P., Behere G.T., Anderson C., Downes S. (2013). A Brave New World for an Old World Pest: *Helicoverpa armigera* (Lepidoptera: Noctuidae) in Brazil. PLoS ONE.

[155] SENAVE (2013). http://www.senave.gov.py/noticias-50-SENAVE-Reportan-presencia-de-peligrosa-plaga-en-nuestro-pais.html.

[156] Murúa M.G., Scalora F.S., Navarro F.R., Cazado L.E., Casmuz A., Villagrán M.E., Lobos E., Gastaminza G. (2014). First Record of *Helicoverpa armigera* (Lepidoptera: Noctuidae) in Argentina. Fla. Entomol..

[157] De Bueno A.F., Sosa-Gómez D.R. (2014). The Old World Bollworm in the Neotropical Region: The experience of Brazilian Growers with *Helicoverpa armigera*. Outlooks Pest Manag..

[158] Coll O.R., Saini E.D. (1992). Insectos y Ácaros Perjudiciales al Cultivo de la Yerba Mate en la Republica Argentina.

[159] Trujillo M.R., Winge H., Ferreira A.G., Mariath J.E.A., Tarasconi L.C. (1995). Agroecosistema yerbatero de alta densidad: Plagas y sus enemigos naturales. Erva-Mate: Biologia e Cultura no Cone Sul.

[160] Alves L.F.A., Santana D.L.Q., Brancalhão R.M.C. (2001). Ocorrência de *Perigonia lusca* (Fabr.) (Lep.: Sphingidae) em Erva-Mate (*Ilex paraguariensis*) no Brasil. Neotrop. Entomol..

[161] Alves L.F.A., Brancalhão R.M.C., Santana D.L.Q. (2001). Ocorrência de Baculovirus em Lagartas de *Perigonia lusca* (Fabr.) (Lep., Sphingidae) no Brasil. Neotrop. Entomol..

[162] Sosa-Gómez D.R., Kitajima E.W., Rolón M. (1994). First record of entomopathogenic diseases in Paraguay tea agroecosystem in Argentina. Fla. Entomol..

[163] Melo F. (2014). Personal Communication.

[164] Sciocco-Cap A., Parola A., Goldberg A., Ghiringhelli D., Romanowski V. (2001). Characterization of a granulovirus (EpapGV) isolated from *Epinotia aporema* (Lepidoptera: Tortricidae) larvae. Appl. Environ. Microbiol..

[165] Goldberg A., Romanowski V., Federici B., Sciocco-Cap A. (2002). Effects of the EpapGV granulovirus on its host, *Epinotia aporema*. J. Invertebr. Pathol..

[166] Ferrelli M.L., Salvador R., Biedma M.E., Berretta M.F., Haase S., Sciocco-Cap A., Ghiringhelli P.D., Romanowski V. (2012). Genome of *Epinotia aporema* granulovirus (EpapGV), a polyorganotropic fast killing betabaculovirus with a novel thymidylate kinase gene. BMC Genomics.

[167] Parola A., Sciocco-Cap A., Glickmann G., Romanowski V. (2003). An immunochemical method for quantification of Epinotia aporema granulovirus (EpapGV). J. Virol. Methods.

[168] Manzan M.A., Biedma M., Aljinovic E.M., Sciocco-Cap A., Romanowski V., Ghiringhelli P.D. (2008). Multiplex PCR and quality control of *Epinotia aporema* Granulovirus (EpapGV) production. Virus Genes.

[169] Moscardi F., Sosa-Gomez D.R., Copping L.G., Green M.B., Rees R.T. (1992). Use of viruses against soybean caterpillars in Brazil. Pest Management in Soybean.

[170] Alexandre T.M., Ribeiro Z.M.A., Craveiro S.R., Cunha F., Fonseca I.C., Moscardi F., Castro M.E. (2010). Evaluation of seven viral isolates as potential biocontrol agents against *Pseudoplusia includens* (Lepidoptera: Noctuidae) caterpillars. J. Invertebr. Pathol..

[171] Craveiro S.R., Melo F.L., Ribeiro Z.M.A., Ribeiro B.M., Báo S.N., Inglis P.W., Castro M.E. (2013). *Pseudoplusia includens* single nucleopolyhedrovirus: Genetic diversity, phylogeny and hypervariability of the pif-2 gene. J. Invertebr. Pathol..

[172] Rodríguez V.A., Belaich M.N., Quintana G., Sciocco-Cap A., Ghiringhelli P.D. (2012). Isolation and Characterization of a Nucleopolyhedrovirus from *Rachiplusia nu* (Guenée) (Lepidoptera: Noctuidae). Int. J. Virol. Mol. Biol..

[173] Arneodo J., Jakubowicz V., Taibo C., Sciocco-Cap A. (2014). Avances en la Caracterización de dos Baculovirus Aislados de la Oruga Medidora Rachiplusia nu (Lepidoptera: Noctuidae).

[174] Castro M.E., Ribeiro Z.M., Santos A.C., Souza M.L., Machado E.B., Sousa N.J., Moscardi F. (2009). Identification of a new nucleopolyhedrovirus from naturally-infected *Condylorrhiza vestigialis* (Guenee) (Lepidoptera: Crambidae) larvae on poplar plantations in South Brazil. J. Invertebr. Pathol..

[175] Williams T., Cisneros J., Caballero P., López Ferber M., Williams T. (2001). Formulación y aplicación de los baculovirus bioinsecticidas. Los Baculovirus y Sus Aplicaciones Como Bioinsecticidas.

[176] Steinke W.E., Giles D.K., Hall F.R., Barry J.W. (1995). Delivery systems for biorational agents. Biorational Pest Control Agents: Formulation and Delivery.

[177] Jones K.A., Westby A., Reilly P.J.A., Jeger M.J., Jones D.G. (1993). The exploitation of microorganisms in the developing countries of the tropics. Exploitation of Micro-Organisms.

[178] Shapiro M., Kurstak E. (1982). *In vivo* mass production of insect viruses. Microbial and Viral Pesticides.

[179] Dulmage H.T., Martínez A.J., Correa J.A. (1970). Recovery of the nuclear polyhedrosis virus of the cabbage looper *Trichoplusia ni* by coprecipitation with lactose. J. Invertebr. Pathol..

[180] Couch T.L., Ignoffo C.M., Burges H.D. (1981). Formulation of insect pathogens. Microbial Control of Insect Pests and Plant Diseases.

[181] Mendugo C.C., Ferraz G., Maia A.H.N., Freitas C.C.L. (1997). Evaluation of a wettable powder formulation for the nuclear polyhedrosis virus of *Anticarsia gemmatalis* (Lep.: Noctuidae). Pestic. Sci..

[182] Smith D.B., Hostetter D.L., Ignoffo C.M. (1978). Formulation and equipment effects on application of a viral (Baculovirus heliothis) insecticide. J. Econ. Entomol..

[183] Ignoffo C.M., García C., Saathoff S.G. (1977). Sunlight stability and rain-fastness of formulations of Baculovirus Heliothis. Environ. Entomol..

[184] Smith D.B., Hostetter D.L., Pinnell R.E., Ignoffo C.M. (1980). Laboratory formulation comparisons for a bacterial (*Bacillus thuringiensis*) and a viral (Baculovirus heliothis) insecticide. J. Econ. Entomol..

[185] Smith D.B., Hostetter D.L., Pinnell R.E., Ignoffo C.M. (1982). Laboratory studies of aerial adjuvants: Formulation development. J. Econ. Entomol..

[186] Henry J.E. (1971). Experimental application of *Nosema locustae* for control of grasshoppers. J. Invertebr. Pathol..

[187] Hostetter D.L., Pinnell R.E. (1983). Laboratory evaluation of plant-derived granules for bollworm control with virus. J. Ga. Entomol. Soc..

[188] Bell M.R., Kranavel R.F. (1978). Tobacco budworm: Development of a spray adjuvant to increase effectiveness of a nuclear polyhedrosis virus. J. Econ. Entomol..

[189] Cañas L.A., O’Neil R.J. (1998). Applications of sugar solutions to maize and the impact of natural enemies on fall armyworm. Int. J. Pest Manag..

[190] Shapiro M. (1992). Use of optical brighteners as radiation protectants for the gypsy moth (Lepidoptera: Lymantriidae) nuclear polyhedrovirus. J. Econ. Entomol..

[191] Wringley G. (1973). Mineral oils as carriers for ultra-low-volume (UVL) spraying. Proc. Natl. Acad. Sci. U.S.A..

[192] Hamm J.J., Shapiro M. (1992). Infectivity of fall armyworm (Lepidoptera: Noctuidae) nuclear polyhedrosis virus enhanced by a fluorescent brightener. J. Econ. Entomol..

[193] Mondragón G., Pineda A., Martínez A., Martínez A.M. (2007). Optical brightener Tinopal C1101 as an ultraviolet protectant for a nucleopolyhedrovirus. Commun. Agric. Biol. Sci..

[194] Martínez A.M., Simón O., Williams T., Caballero P. (2003). Effect of optical brighteners on the insecticidal activity of a nucleopolyhedrovirus in three instars of *Spodoptera frugiperda* (Lepidoptera: Noctuidae). Entomol. Exp. Appl..

[195] Washburn J.O., Kirkpatrick B.A., Haas-Stapleton E., Volkman L.E. (1998). Evidence that the stilbene-derived optical brightener M2R enhances *Autographa californica* M nucleopolyhedrovirus infection of *Trichoplusia ni* and *Heliothis virescens* by preventing sloughing of infected midgut epithelial cells. Biol. Control.

[196] Wang P., Granados R. (2000). Calcofluor disrupts the midgut defense system in insects. Insect Biochem. Mol. Biol..

[197] Morales L., Moscardi F., Sosa-Gómez D.R., Paro F.E., Soldorio I.L. (1997). Enhanced activity of *Anticarsia gemmatalis* Hüb. (Lepidoptera: Noctuidae) nuclear polyhedrosis virus by boric acid in the laboratory. An. Soc. Entomol. Bras..

[198] Cisneros J., Pérez J.A., Penagos D.I., Ruiz J., Goulson D., Caballero P., Cave R.D., Williams T. (2002). Formulation of a nucleopolyhedrovirus with boric acid for control of *Spodoptera frugiperda* (Lepidoptera: Noctuidae) in maize. Biol. Control.

[199] Ignoffo C.M., Shasha B.S., Shapiro M. (1991). Sunlight ultraviolet protection of the Heliothis nuclear polyhedrosis virus through starch-encapsulation technology. J. Invertebr. Pathol..

[200] Morales-Ramos L.H., McGuire M.R., Galán-Wong L.J. (1998). Utilization of several biopolymers of granular formulations of *Bacillus thuringiensis*. J. Econ. Entomol..

[201] Morales-Ramos L.H., McGuire M.R., Galán-Wong L.J., Franco-Castro R. (2000). Evaluation of pectin, gelatin, and starch granular formulations of *Bacillus thuringiensis*. Southwest. Entomol..

[202] Villamizar L., Barrera G., Cotes A.M., Martínez F. (2010). Eudragit S100 microparticles containing *Spodoptera frugiperda* nucleopolyehedrovirus: Physicochemical characterization, photostability and *in vitro* virus release. J. Microencapsul..

[203] Tamez-Guerra P., McGuire M.R., Behle R.W., Hamm J.J., Sumner H.R., Shasha B.S. (2000). Sunlight persistence and rainfastness of spray-dried formulations of the *Anagrapha falcifera* baculovirus. J. Econ. Entomol..

[204] Gioria V., Beccaría A., Claus J.D. (2006). Crecimiento, metabolismo y producción de baculovirus en cultivos en suspensión de una línea celular del insecto lepidóptero *Anticarsia gemmatalis*. Quim. Viva.

[205] Claus J.D., Remondetto G., Guerrero S., Demonte A., Murguía M., Mancipar A. (1993). *Anticarsia gemmatalis* nuclear polyhedrosis virus replication in serum-free and serum-reduced insect cell cultures. J. Biotechnol..

[206] Micheloud G.A., Gioria V.V., Pérez G., Claus J.D. (2009). Production of occlusion bodies of *Anticarsia gemmatalis multiple nucleopolyhedrovirus* in serum-free suspension cultures of the saUFL-AG-286 cell line: Influence of infection conditions and statistical optimization. J. Virol. Methods.

[207] Shuler M.A., Kargi F. (2006). Bioprocess Engineering—Basic Concepts.

[208] Micheloud G.A., Gioria V.V., Eberhardt I., Visnovsky G., Claus J.D. (2011). Production of the Anticarsia gemmatalis multiple nucleopolyhedrovirus in serum-free suspension cultures of the saUFL-AG-286 cell line in stirred reactor and airlift reactor. J. Virol. Methods.

[209] Claus J.D., Gioria V.V., Micheloud G.A., Visnovsky G., Soloneski S., Larramendy M. (2012). Production of Insecticidal Baculoviruses in Insect Cell Cultures: Potential and Limitations. Insecticides—Basic and Other Applications.

[210] Van Beek N., Davis D.C. (2014). Baculovirus insecticide production in insect larvae. Methods Mol. Biol..

[211] Keeley L.L., Hayes T.K. (1987). Speculations on biotechnology applications for insect neuroendocrine research. Insect Biochem..

[212] Maeda S. (1989). Increased insecticidal effect by a recombinant baculovirus carrying a synthetic diuretic hormone gene. Biochem. Biophys. Res. Commun..

[213] Menn J.J., Borkovec A.B. (1989). Insect neuropeptides—Potential new insect control agents. J. Agric. Food Chem..

[214] Carbonell L.F., Hodge M.R., Tomalski M.D., Miller L.K. (1988). Synthesis of a gene coding for an insect specific scorpion neurotoxin and attempts to express it using baculovirus vectors. Gene.

[215] Kroemer J.A., Bonning B.C., Harrison R.L. (2015). Expression, Delivery and Function of Insecticidal Proteins Expressed by Recombinant Baculoviruses. Viruses.

[216] Bonning B.C., Hammock B.D. (1996). Development of recombinant baculoviruses for insect control. Annu. Rev. Entomol..

[217] Wood H.A., Gunasekaran M., Weber D.J. (1996). Genetically enhanced baculovirus insecticides. Molecular Biology of the Biological Control of Pests and Diseases of Plants.

[218] Coast G.M., Orchard I., Phillips J.E., Schooley D.A. (2002). Insect diuretic and antidiuretic hormones. Adv. Insect Physiol..

[219] Gade G. (2004). Regulation of intermediary metabolism and water balance of insects by neuropeptides. Annu. Rev. Entomol..

[220] Holman G.M., Nachman R.J., Wright M.S. (1990). Insect neuropeptides. Annu. Rev. Entomol..

[221] O’Reilly D.R. (1995). Baculovirus-encoded ecdysteroid UDP-glucosyltransferases. Insect Biochem. Mol. Biol..

[222] O’Reilly D.R., Miller L.K. (1989). A baculovirus blocks insect molting by producing ecdysteroid UDP-glucosyltransferase. Science.

[223] Slavicek J.M., Adoga M.P. (2012). Baculovirus Enhancins and Their Role in Viral Pathogenicity. Molecular Virology.

[224] Arana E.I., Albariño C.G., O’Reilly D., Ghiringhelli P.D., Romanowski V. (2001). Generation of a recombinant *Anticarsia gemmatalis multicapsid nucleopolyhedrovirus* expressing a foreign gene under the control of a very late promoter. Virus Genes.

[225] Ribeiro B.M., Gatti C.D., Costa M.H., Moscardi F., Maruniak J.E., Possee R.D., Zanotto P.M. (2001). Construction of a recombinant *Anticarsia gemmatalis nucleopolyhedrovirus* (AgMNPV-2D) harbouring the beta-galactosidase gene. Arch. Virol..

[226] Pinedo F.J.R., Moscardi F., Luque T., Olszewski J.A., Ribeiro B.M. (2003). Inactivation of the ecdysteroid UDP-glucosyltransferase (egt) gene of *Anticarsia gemmatalis* nucleopolyhedrovirus (AgMNPV) improves its virulence towards its insect host. Biol. Control.

[227] Haase S., McCarthy C.B., Ferrelli M.L., Pidre M.L., Sciocco-Cap A., Romanowski V. (2015). Development of a recombination system for the generation of occlusion positive genetically modified *Anticarsia gemmatalis Multiple Nucleopolyhedrovirus*. Viruses.

[228] Haase S., López M.G., Jaramillo C., Sciocco-Cap A., Taboga O., Romanowski V. Study of polyhedrin functional complementation among nucleopolyhedroviruses.

[229] Inceoglu A.B., Kamita S.G., Hammock B.D. (2006). Genetically modified baculoviruses: A historical overview and future outlook. Adv. Virus Res..

[230] Wood H.A., Hughes P.R., Shelton A. (1994). Field studies of the co-occlusion strategy with a genetically altered isolate of the *Autographa californica* nuclear polyhedrosis virus. Environ. Entomol..

[231] Lima A.A., Aragao C.W., de Castro M.E., Oliveira J.V., Sosa Gomez D.R., Ribeiro B.M. (2013). A recombinant *Anticarsia gemmatalis* MNPV harboring chiA and v-cath genes from *Choristoneura fumiferana* defective NPV induce host liquefaction and increased insecticidal activity. PLoS ONE.

[232] Simón O., Williams T., Asensio A.C., Ros S., Gaya A., Caballero P., Possee R.D. (2008). Sf29 gene of *Spodoptera frugiperda multiple nucleopolyhedrovirus* is a viral factor that determines the number of virions in occlusion bodies. J. Virol..

[233] Bonning B.C., Boughton A.J., Jin H., Harrison R.L., Upadhyay K. (2002). Genetic enhancement of baculovirus insecticides. Advances in Microbial Control of Insect Pests.

[234] Cory J.S., Hirst M.L., Williams T., Halls R.S., Goulson D., Green B.M., Carty T.M., Possee R.D., Cayley P.J., Bishop D.H.L. (1994). Field trial of a genetically improved baculovirus insecticide. Nature (Lond.).

[235] Ardisson-Araújo D.M., Melo F.L., de Souza Andrade M., Brancalhão R.M.C., Báo S.N., Ribeiro B.M. (2014). Complete genome sequence of the first non-Asian isolate of *Bombyx mori* nucleopolyhedrovirus. Virus Genes.

[236] Oliveira J.V.C., Wolf J.L.C., Garcia-Muriak A., Ribeiro B.M., de Castro M.E., de Souza M.L., Moscardi F., Maruniak J.E., Zanotto P.M. (2006). Genome of the most widely used viral biopesticide: *Anticarsia gemmatalis* multiple nucleopolyhedrovirus. J. Gen. Virol..

[237] Wolff J.L., Valicente F.H., Martins R., Oliveira J.V., Zanotto P.M. (2008). Analysis of the genome of *Spodoptera frugiperda* nucleopolyhedrovirus (SfMNPV-19) and of the high genomic heterogeneity in group II nucleopolyhedroviruses. J. Gen. Virol..

[238] Meister G., Tuschl T. (2004). Mechanisms of gene silencing by double-stranded RNA. Nature.

[239] Sander J.D., Joung J.K. (2014). CRISPR-Cas systems for editing, regulating and targeting genomes. Nat. Biotechnol..

[240] Herbert T., Vonada R., Jenkins M., Byon R., Frausto Leyva J.M. (2010). Environmental Funds and Payments for Ecosystems Services: RedLAC Capacity Building Project for Environmental Funds.

